# Supplementary material for: A Comparative Study of Serum Pharmacochemistry of Kai-Xin-San in Normal and AD Rats Using UPLC-LTQ-Orbitrap-MS
Source: Pharmaceuticals (Basel). 2022 Dec 26;16(1):30. doi: 10.3390/ph16010030 (PMC9866203; doi:10.3390/ph16010030)
Supplement: Supplementary file 1 [file pharmaceuticals-16-00030-s001.zip › pharmaceuticals-2077681-supplementary.pdf]

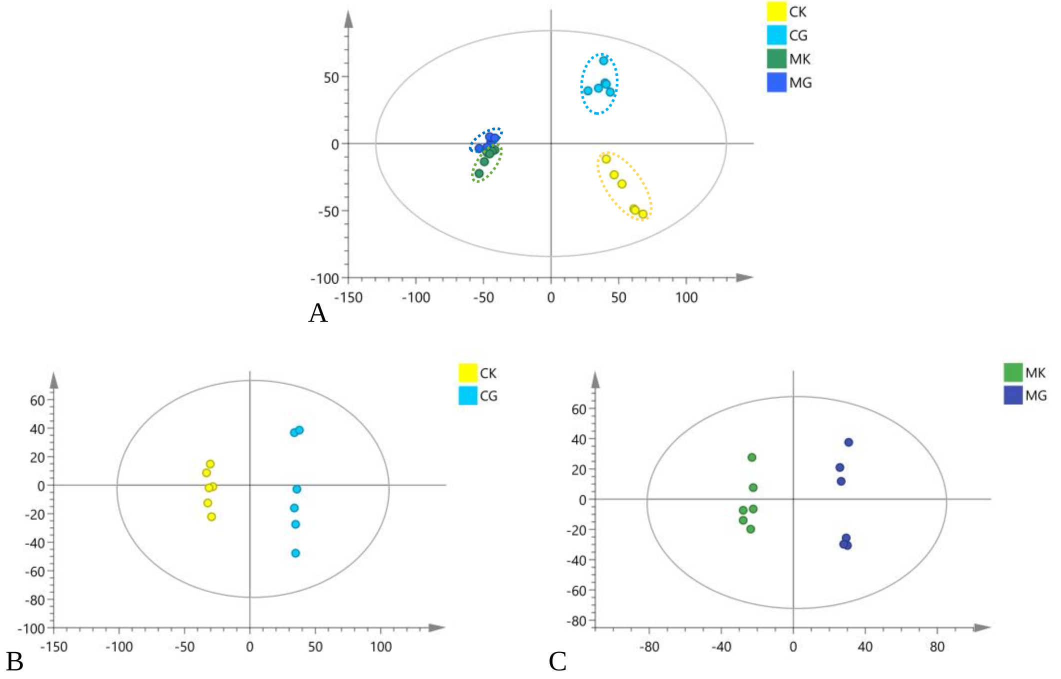

Figure S1 Multivariate statistical analysis of serum metabolic profile in positive ion mode. A, PCA plot of CK, CG, MK and MG groups; B, OPLS-DA plot of CK and CG groups; C, OPLS-DA plot of MK and MG groups.

Table S1 The identified main components of KXS and their relative information

No.	Compound	t_R/min	Fomula	Measured	Mass error/ppm	Ion Addition	Fragment ions (m/z)
1	L(+)-Arginine	0.86	$C_6H_{14}N_4O_2$	175.1119	4.6	$[M+H]^-$	158.0920,130.0971,116.0702,112.0865
2	Sucrose	4.07	$C_{12}H_{22}O_{11}$	341.1142	-4.1	$[M-H]^-$	179.0585,161.0478,119.0370,113.0260,89.0258
3	Sibiricose A3	4.15	$C_{19}H_{26}O_{13}$	461.136	-1.5	$[M-H]^-$	299.0809,281.0701,239.0591,209.0482,179.0374,137.0263
4	Sibiricose A5	5.78	$C_{22}H_{30}O_{14}$	517.1624	-1.2	$[M-H]^-$	341.1133,337.0973,193.0528,175.0425
5	Sibiricose A6	6.15	$C_{23}H_{32}O_{15}$	547.1743	1.5	$[M-H]^-$	367.1097,341.1147,223.0632,205.0542,190.0298
6	Sibiricose A1	6.46	$C_{23}H_{32}O_{15}$	547.1737	0.4	$[M-H]^-$	367.1099,341.1150,223.0634,205.0544,190.0296
7	Tatarine C	7.74	$C_{15}H_{20}N_2O_6$	323.1299	-4.9	$[M-H]^-$	305.1125,203.0817,189.0661,185.0085,175.0870
8	2,5-dimethoxy benzoquinone	7.95	$C_8H_8O_4$	169.0422	3.0	$[M+H]^-$	151.1116,141.0546,125.0595,123.0802,109.1009
9	Acoramol	8.73	$C_{12}H_{16}O_4$	225.1047	1.8	$[M+H]^-$	210.0908,207.1036,193.0878,168.0797
10	Tataroside	9.17	$C_{26}H_{44}O_{12}$	547.2839	2.2	$[M-H]^-$	529.2719,509.2475,385.2301,367.2193,349.2084,319.1973,301.18 62
11	Sibiricaxanthone A	9.7	$C_{24}H_{26}O_{14}$	537.1311	-1.1	$[M-H]^-$	417.0883,387.0773,315.0552,285.0443,267.0335
12	Polygalaxanthone VI	10.64	$C_{23}H_{26}O_{12}$	493.1355	0.8	$[M-H]^-$	317.0634, 302.0456, 175.0561
13	2,4,5- Trimethoxybenzoic acid	10.68	$C_{10}H_{12}O_5$	213.0669	-4.7	$[M+H]^-$	195.0650,165.0004
14	PolygalaxanthoneIII	10.85	$C_{25}H_{28}O_{15}$	567.1432	1.7	$[M-H]^-$	447.0994,417.0884,399.0775,345.0663,315.0554,272.0364
15	Ginsenjilinol/Majonoside R_1	10.89	$C_{42}H_{72}O_{15}$	861.4854	-2.1	$[M-H+HCOO]^-$	815.4890,653.4346,635.4238,553.3450,491.3822,391.2922
16	Sibiricose A2	12.3	$C_{24}H_{34}O_{15}$	561.1884	-1.4	$[M-H]^-$	323.1041,279.0920,237.0797
17	Lancerin	12.4	$C_{19}H_{18}O_{10}$	405.0882	-2.9	$[M-H]^-$	315.0492,297.0388,285.0389,257.0444,243.1227
18	Tenuifoliside B	12.58	$C_{30}H_{36}O_{17}$	667.1955	1.1	$[M-H]^-$	461.1363,281.0704,239.0579,205.0534
19	Tatarinoids A	13.98	$C_{12}H_{16}O_5$	241.0989	-1.2	$[M+H]^-$	223.0959,215.2825,195.1012,169.0856,154.0621
20	Acoramone	14	$C_{12}H_{16}O_4$	225.1039	-1.7	$[M+H]^-$	210.0909,207.1038,193.0879,168.0799
21	3,6'-Disinapoyl sucrose	14.15	$C_{34}H_{42}O_{19}$	753.2334	2.6	$[M-H]^-$	547.1742,529.1635,367.1100,265.0752,223.0634
22	Ginsenjilinol/Majonoside R_1	14.19	$C_{42}H_{72}O_{15}$	861.4896	3.1	$[M-H+HCOO]^-$	815.4736,653.4221,553.3343,491.0968

23	Sibiricose A4	14.66	$C_{34}H_{42}O_{19}$	753.2335	2.7	$[M-H]^-$	547.1734,529.1627,367.1100,265.0758,223.0634
24	(Z)-Coniferyl alcohol	14.69	$C_{10}H_{12}O_3$	181.0777	-1.6	$[M+H]^-$	151.0769,148.0534,138.0689,121.0660,107.9603
25	Tenuifoliside A	14.79	$C_{31}H_{38}O_{17}$	681.2106	4.3	$[M-H]^-$	635.2631,473.1381,443.1272,281.0714,239.0592,223.0635
26	Notoginsenoside N	15.11	$C_{48}H_{82}O_{19}$	1007.5436	-8.3	$[M-H+HCOO]^-$	799.4949,637.4404,475.3856,391.2919
27	2,4,5-Trimethoxybenzaldehyde	15.19	$C_{10}H_{12}O_4$	197.0726	-2.0	$[M+H]^-$	182.0570,169.0855,154.0621,138.0673
28	Asaronaldehyde	15.21	$C_{10}H_{12}O_4$	197.0728	-1.0	$[M+H]^-$	182.0571,169.0856,154.0622,138.0673
29	20-O-Glucosylginsenoside Rf	15.66	$C_{48}H_{82}O_{19}$	1007.5433	-1.1	$[M-H+HCOO]^-$	799.4956,637.4409,475.3860,391.2922
30	Reiniose B	15.99	$C_{29}H_{34}O_{15}$	621.1881	-1.7	$[M-H]^-$	499.1422,445.1322,337.0908,193.0498,175.0394
31	Notoginsenoside Fp_1	16.24	$C_{47}H_{80}O_{18}$	977.5321	-1.9	$[M-H+HCOO]^-$	799.4938,637.4397,475.3869,391.2921
32	Tenuifoliside W	16.38	$C_{67}H_{86}O_{39}$	1513.4674	-4.3	$[M-H]^-$	1367.3986,1325.3891,1191.3531,1149.3431,1131.3330,1069.3175
33	Ginsenoside Rg_1	17.37	$C_{42}H_{72}O_{14}$	845.4935	2.3	$[M-H+HCOO]^-$	799.4956,637.4409,619.4301,475.3861
34	Ginsenoside Re	17.43	$C_{48}H_{82}O_{18}$	991.5533	3.3	$[M-H+HCOO]^-$	945.5263,799.4943,783.4994,637.4403,475.3876,391.2927
35	Tenuifoliside V	17.79	$C_{53}H_{70}O_{33}$	1233.3811	1.4	$[M-H]^-$	1191.3524,1173.3422,1111.3266,1057.3166,1015.3064,997.2962,935.2810,893.2808,731.2196,527.1578
36	Tenuifoliose G	17.82	$C_{66}H_{84}O_{38}$	1483.4735	6.7	$[M-H]^-$	1337.4143,1215.3769,1161.3658,1143.3553,1119.3548,1101.3441,1039.3276,997.3165
37	Tenuifoliose M	17.83	$C_{65}H_{82}O_{37}$	1453.4636	7.3	$[M-H]^-$	1337.4144,1215.3772,1161.3659,1143.3555,1101.3443,1039.3280,997.3169
38	Tenuifoliose L	18.12	$C_{67}H_{84}O_{38}$	1495.4753	7.8	$[M-H]^-$	1349.4150,1307.4043,1203.3768,1185.3665,1161.3662,1143.3555,1081.3391,1039.3282,795.2454
39	Tenuifoliose F	18.3	$C_{68}H_{86}O_{39}$	1525.4795	3.6	$[M-H]^-$	1379.4001,1337.3901,1319.3798,1203.3546,1161.3442,1143.3337
40	Tenuifoliside C	18.34	$C_{35}H_{44}O_{19}$	767.2484	1.6	$[M-H]^-$	529.1628,367.1100,325.0986,265.0757,223.0634
41	Tenuifoliose K	18.35	$C_{57}H_{70}O_{32}$	1265.3925	6.3	$[M-H]^-$	1119.3546,1101.3449,1077.3443,1059.3337,997.3170,973.3169,915.2893,835.2625,753.2348
42	Isocalamediol	18.73	$C_{15}H_{22}O_3$	251.1573	3.9	$[M+H]^-$	233.1534,215.1429,205.1586,191.1429,173.1324
43	Tenuifoliose T	18.76	$C_{56}H_{70}O_{32}$	1253.3884	3.1	$[M-H]^-$	1131.3531,1107.3531,1077.3421,985.3151,929.2827,823.2608

44	Tenuifoliose P	18.8	$C_{59}H_{74}O_{34}$	1325.4141	6.4	$[M-H]^-$	1203.3754,1119.3532,1077.3424,997.3155,753.2337
45	Tenuifoliose E	18.82	$C_{58}H_{72}O_{33}$	1295.3989	3.0	$[M-H]^-$	1119.3542,1077.3436,997.3165,979.3058,753.2347
46	isoacoramone	19.07	$C_{12}H_{16}O_4$	225.1036	-3.1	$[M+H]^-$	210.0883,193.0855,165.0908
47	Tenuifoliside S	19.43	$C_{55}H_{68}O_{31}$	1223.3787	3.9	$[M-H]^-$	1101.3437,1077.3437,1061.3275,955.3054,899.2728,793.2511,613 .1856
48	Tenuifoliose X	19.64	$C_{57}H_{72}O_{33}$	1283.4029	6.1	$[M-H]^-$	1161.3653,1131.3544,1107.3544,1089.3438,985.3162,959.2944,82 3.2618
49	(E)-3-(2,4,5- Trimethoxyphenyl) acrylaldehyde	19.98	$C_{12}H_{14}O_4$	223.0879	-3.1	$[M+H]^-$	208.0727,195.1011,167.0700,165.0907
50	Polygalasaponin XXIX	20.3	$C_{64}H_{102}O_{33}$	1397.6297	-5.3	$[M-H]^-$	1367.5941, 1143.5597, 717.2361, 455.3165, 425.3011
51	Tatarine A	20.83	$C_{17}H_{13}NO_3$	280.0882	-2.5	$[M+H]^-$	280.0965,265.0730,252.0653,236.0704
52	Tenuifoliose B	21	$C_{60}H_{74}O_{34}$	1337.4129	5.4	$[M-H]^-$	1295.4021,1215.3751,1161.3638,1143.3532,1119.3530,1101.3425, 1039.3262,997.3151,753.2330
53	Pseudoginsenoside Rt_3	21.06	$C_{41}H_{70}O_{13}$	815.4845	4.5	$[M-H+HCOO]^-$	769.4845,637.4408,619.4300,475.3858,
54	Desacylsenegasaponin B	21.08	$C_{59}H_{94}O_{29}$	1265.5754	-9.5	$[M-H]^-$	1235.5620,585.2000,499.1638,455.3140,425.3035
55	Onjisaponin Pg	21.19	$C_{70}H_{110}O_{37}$	1541.6811	5.8	$[M-H]^-$	1397.6370,1367.6276,1173.5828,455.3243
56	2,3,3a,7,8,8a-Hexahydro3a-hydroxy-1,4 -dimethyl7-(1-methylethylidene)- 6(1H)-azulenone	21.2	$C_{15}H_{22}O_2$	235.1626	5.1	$[M+H]^-$	217.1585,207.1378,193.1584,175.1480,161.1324,133.1010,119.08 54
57	Tenuifoliose C	21.4	$C_{58}H_{72}O_{33}$	1295.4033	6.4	$[M-H]^-$	1173.3656,1119.3541,1101.3441,1077.3436,1059.3328, 997.3165,851.2782,753.2347
58	Floralginsenoside B	21.71	$C_{41}H_{70}O_{13}$	815.4823	1.7	$[M-H+HCOO]^-$	769.4688,637.4276,475.3761,391.2830
59	Notoginsenoside Rt	21.9	$C_{44}H_{74}O_{15}$	887.4989	-4.0	$[M-H+HCOO]^-$	841.4894,799.4790,781.4690,637.4278,619.4175,475.3763,391.28 31
60	Tenuifoliose O	21.93	$C_{61}H_{76}O_{35}$	1367.4178	1.2	$[M-H]^-$	1191.3748,1149.3641,1131.3532,425.3133
61	Tataramide B	22.13	$C_{36}H_{36}N_2O_8$	625.2433	-5.2	$[M-H]^-$	488.1698,462.1909,364.1177,325.1069

62	Polygalasaponin XXII	22.19	$C_{58}H_{92}O_{28}$	1236.5847	9.3	$[M-H]^-$	1205.5728,1011.5285,981.5175,455.3247,425.3138
63	6′-acetyl-ginsenoside Rg_1	22.2	$C_{44}H_{74}O_{15}$	887.4952	-8.3	$[M-H+HCOO]^-$	841.4876,799.4772,781.4637,637.4263,619.4161,475.3754,391.2825
64	OnjisaponinQg	22.25	$C_{76}H_{120}O_{41}$	1687.7419	7.1	$[M-H]^-$	1585.7100,1543.6989,1513.6892,1411.6561
65	Polygalasaponin XXIII	22.46	$C_{53}H_{82}O_{24}$	1101.5169	-1.9	$[M-H]^-$	1071.4942,1059.3133,453.2982,423.2879
66	Polygalasaponin XXVIII	22.93	$C_{53}H_{84}O_{24}$	1103..5416	6.2	$[M-H]^-$	1073.5290,455.3241,425.3132
67	Tenuifoliose D	23.07	$C_{60}H_{74}O_{34}$	1337.3996	-4.5	$[M-H]^-$	1295.3786,1215.3531,1161.3427,1143.3327,1119.3327,1101.3221, 1039.3070,997.2967,
68	Tenuifoliose H	23.53	$C_{61}H_{74}O_{34}$	1349.4042	-1.0	$[M-H]^-$	1203.3753,1161.3647,1143.3543,1081.3377,1039.3269
69	Onjisaponin TG	23.68	$C_{64}H_{100}O_{32}$	1379.6145	-3.4	$[M-H]^-$	1317.6220,1277.5892,1235.5848,1205.5677,455.3267
70	Tenuifoliose J	23.69	$C_{59}H_{72}O_{33}$	1307.4036	6.5	$[M-H]^-$	1265.3927,1161.3656,1143.3548,1131.3547,1119.3547, 1039.3276,997.3167
71	Onjisaponin TF	23.77	$C_{59}H_{94}O_{28}$	1249.5994	5.4	$[M-H]^-$	1219.5903,1025.5456,995.5347,455.3229,425.3119
72	Reinioside F	23.98	$C_{69}H_{108}O_{36}$	1511.6691	5.1	$[M-H]^-$	1367.6285,1337.6186,1293.4099,1143.5737,455.3231
73	Onjisaponin TE	24.28	$C_{70}H_{110}O_{36}$	1525.6882	7.2	$[M-H]^-$	1423.6532,1381.6418,1351.6320,1157.5872,455.3243
74	Tenuifoliose A	25.16	$C_{62}H_{76}O_{35}$	1379.4038	-8.9	$[M-H]^-$	1337.4094,1203.3698,1161.3585,1143.3480,1081.3319, 1039.3201
75	Tenuifoliose N	25.61	$C_{63}H_{78}O_{36}$	1409.4351	5.9	$[M-H]^-$	1233.3870,1215.3772,1191.3765,1173.3662,1111.3496, 1069.3385,825.2564
76	Ginsenoside Rf	25.68	$C_{42}H_{72}O_{14}$	845.4854	-7.7	$[M-H+HCOO]^-$	799.4787,637.4279,475.3762
77	β-Asarone	28.13	$C_{12}H_{16}O_3$	209.1088	-2.4	$[M+H]^-$	208.0754,194.0935,181.0857,162.0674,149.0960,121.0646
78	α-Asarone	28.14	$C_{12}H_{16}O_3$	209.1081	-5.4	$[M+H]^-$	208.0755,194.0935,181.0857,162.0674,149.0959,121.0646
79	Tenuifolin	28.21	$C_{36}H_{56}O_{12}$	679.3767	1.4	$[M-H]^-$	455.3245,425.3136
80	Notoginsenoside Fa	28.38	$C_{59}H_{100}O_{27}$	1239.6489	3.4	$[M-H+HCOO]^-$	1107.6090,1077.5988,945.5547,783.5003,621.4455, 459.3906
81	Ligraminol C	29.32	$C_{23}H_{28}O_6$	401.1851	-7.2	$[M+H]^-$	383.1846,371.1847,263.1274,233.1169,218.0934,195.1013,181.0856,151.0751
82	Ginsenoside Rg_2	29.5	$C_{42}H_{72}O_{13}$	829.4908	-8.1	$[M-H+HCOO]^-$	783.4700,637.4271,475.3758,391.2828

83	Ginsenoside Rh_1	29.73	$C_{36}H_{62}O_9$	683.4379	-1.4	$[M-H+HCOO]^-$	637.4328,475.3793
84	Ginsenoside Ra_2	30.76	$C_{58}H_{98}O_{26}$	1209.6383	3.5	$[M-H+HCOO]^-$	1077.5980,945.5546,783.5001,621.4455,459.3906, 375.2972
85	Ginsenoside Rb_1	31.74	$C_{54}H_{92}O_{23}$	1153.6052	2.6	$[M-H+HCOO]^-$	1107.6075,945.5536,783.4993,621.4449,459.3925, 375.2978
86	Ligraminol D	32	$C_{21}H_{28}O_6$	375.1893	3.4	$[M+H]^-$	357.1787,295.2317,277.2209,263.0993,191.0773,183.1416
87	Ginsenoside Ro	32.59	$C_{48}H_{76}O_{19}$	955.4961	-2.0	$[M-H]^-$	793.4325,731.4329,613.3706,569.3811,523.3759,455.3503
88	Ginsenoside Ra_1	33.5	$C_{58}H_{98}O_{26}$	1209.6397	4.2	$[M-H+HCOO]^-$	1077.5986,945.5553,915.5445,783.5006,621.4459, 459.3911
89	Ginsenoside F_1	35.1	$C_{36}H_{62}O_9$	683.4337	-8.7	$[M-H+HCOO]^-$	637.4274,475.3761,391.2832
90	Ginsenoside Rb_2	35.13	$C_{53}H_{90}O_{22}$	1123.5988	6.4	$[M-H+HCOO]^-$	1077.5937,945.5550,783.5004,621.4458,459.3908, 375.2970
91	Chikusetsusaponin Iv	36.23	$C_{47}H_{74}O_{18}$	925.4904	3.1	$[M-H+HCOO]^-$	793.4323,613.3702,569.3808,455.3503
92	1,6-Dihydroxy-3,5,7-trimethoxyxanthon e	37.06	$C_{16}H_{14}O_7$	317.0705	-9.1	$[M-H]^-$	302.0471,287.0238,
93	Quinquenoside R_1	37.64	$C_{56}H_{94}O_{24}$	1149.6098	-3.2	$[M-H+HCOO]^-$	1107.5863,1059.5667,945.5354,783.4842,621.4325, 459.3810
94	Chikusetsusaponin Iva	38.2	$C_{42}H_{66}O_{14}$	793.4487	5.0	$[M-H+HCOO]^-$	793.3949,631.3805,613.3705,569.3808,455.3501
95	Ginsenoside Rd	39.22	$C_{48}H_{82}O_{18}$	991.558	8.9	$[M-H+HCOO]^-$	945.5084,783.5003,621.4458,459.3908,375.2970
96	Ginsenoside Rs_1/Rs_2/isomer	40.17	$C_{55}H_{92}O_{23}$	1165.6135	9.4	$[M-H+HCOO]^-$	1077.5979,1059.5882,945.5550,783.5004,621.4456, 459.3923
97	Isoeugenol	40.32	$C_{11}H_{14}O_2$	179.0978	-5.6	$[M+H]^-$	164.0783,151.0750,121.0645,91.0539
98	Malonyl-Rd	41.76	$C_{51}H_{84}O_{21}$	1031.5549	4.8	$[M-H+HCOO]^-$	987.5645,945.5505,459.5828
99	Ginsenoside Rs_1/Rs_2/isomer	42.44	$C_{55}H_{92}O_{23}$	1165.6052	2.0	$[M-H+HCOO]^-$	1077.5982,1059.5883,945.5553,783.5006,621.4461, 459.3923
100	OnjisaponinFg	42.47	$C_{81}H_{120}O_{40}$	1731.7378	1.6	$[M-H]^-$	1701.7386,1669.7474,1629.7157,1599.7054,1587.7045,1557.6939,
101	Ginsenoside Rs_1/Rs_2/isomer	43.24	$C_{55}H_{92}O_{23}$	1165.6045	1.4	$[M-H+HCOO]^-$	1077.5969,1059.5866,945.5538,783.4991,621.4453
102	Ginsenoside Ra_6	44.5	$C_{58}H_{96}O_{24}$	1221.6218	-6.2	$[M-H+HCOO]^-$	1175.5556,1107.6091,1089.5992,945.5550,783.5004, 621.4462,
103	Onjisaponin F	45.03	$C_{75}H_{112}O_{36}$	1587.6789	-8.6	$[M-H]^-$	1587.6463,1557.6364,567.1937
104	Onjisaponin TH	45.36	$C_{65}H_{96}O_{28}$	1323.6173	6.8	$[M-H]^-$	1293.6066,455.3230,425.3134
105	Onjisaponin L	45.98	$C_{86}H_{128}O_{43}$	1847.7707	-6.2	$[M-H]^-$	1847.7648,1785.7613,1703.7221,1673.7072,
106	Ginsenoside Rd_2/Notoginsenoside Fe	46.54	$C_{47}H_{80}O_{17}$	961.5406	1.2	$[M-H+HCOO]^-$	915.5426,783.4992,621.4448,
107	γ-Asarone	46.76	$C_{12}H_{16}O_3$	209.1081	-5.7	$[M+H]^-$	194.0936,181.0858,162.0675,149.0960,121.0647

108	Ginsenoside Rd2/Notoginsenoside Fe	47.54	$C_{47}H_{80}O_{17}$	961.5364	-3.3	$[M-H+HCOO]^-$	915.5249,783.4828,459.5309
109	Onjisaponin R	47.69	$C_{76}H_{114}O_{37}$	1617.6912	-7.4	$[M-H]^-$	1617.7119,1587.7009,1155.5672,1125.5544,699.2368,613.1994,455.3162
110	Onjisaponin B	48	$C_{75}H_{112}O_{35}$	1571.6915	-4.1	$[M-H]^-$	1571.7065,1541.6960,1317.6380,567.1940,455.3168
111	Onjisaponin V	49.46	$C_{82}H_{122}O_{41}$	1761.7345	-6.3	$[M-H]^-$	1761.7580,1699.7570,1659.7247,1617.7113,1587.7023
112	Onjisaponin H	49.88	$C_{74}H_{110}O_{34}$	1541.672	-9.9	$[M-H]^-$	1541.6706,1511.6602,,1317.6185,1287.6085,1139.5572, 537.1791,455.3119
113	Polygalasaponin XXXII	50.09	$C_{79}H_{118}O_{38}$	1673.7166	-7.7	$[M-H]^-$	1673.7377,1643.7293,1449.6823,1419.6711,1287.6262, 669.2264,537.1830
114	Onjisaponin E	50.62	$C_{71}H_{106}O_{33}$	1485.6474	-9.1	$[M-H]^-$	1485.6682,1455.6572,675.3919,455.3170,425.3063
115	Onjisaponin Z	51.1	$C_{71}H_{106}O_{32}$	1469.6528	-9.0	$[M-H]^-$	1469.6726,1439.6610,1245.6153,1215.6043,1007.5269, 953.4944,455.3169,425.3063
116	Onjisaponin Ng	51.38	$C_{80}H_{118}O_{38}$	1685.7187	-6.4	$[M-H]^-$	1685.7398,1583.7063,1541.6928,1317.6375,537.1826
117	1-(4-Methoxyphenyl)allyl acetate	51.61	$C_{12}H_{14}O_3$	207.0922	-7.2	$[M+H]^-$	176.0830,151.0753,133.0647
118	Onjisaponin G	52.55	$C_{70}H_{104}O_{32}$	1455.6534	1.9	$[M-H]^-$	1455.6324,1425.6491,993.4977,963.4878,537.1776, 455.3132,425.3029
119	Onjisaponin Y	53.37	$C_{69}H_{102}O_{30}$	1409.6325	-8.8	$[M-H]^-$	1409.6258,1379.6146,1277.5839,1247.5737,1185.5737, 455.3132,425.30298
120	Onjisaponin W	54.06	$C_{81}H_{120}O_{40}$	1731.7237	-6.5	$[M-H]^-$	1731.6851,1669.6867,1629.6564,1587.6474,1557.6385, 669.2263
121	Onjisaponin S	54.15	$C_{81}H_{122}O_{40}$	1733.7487	-1.0	$[M-H]^-$	1733.7534,1670.7480,1630.7152,1588.7031,1558.6937
122	Ginsenoside F_2	56.02	$C_{42}H_{72}O_{13}$	829.4897	-8.9	$[M-H+HCOO]^-$	783.4848,621.4332,459.3817
123	Bu-Gypenoside XVI	56.13	$C_{52}H_{86}O_{19}$	1059.5688	-7.3	$[M-H+HCOO]^-$	1013.5601,945.5060,927.5257,783.4837,765.4736, 621.4328,459.3807
124	Onjisaponin Gg	56.2	$C_{76}H_{112}O_{36}$	1599.7023	6.0	$[M-H]^-$	1569.6940,1497.6727,1455.6623,1425.6517,1155.5739, 537.1901,455.3231
125	Onjisaponin MF	57.13	$C_{63}H_{92}O_{26}$	1263.5839	-2.5	$[M-H]^-$	1233.5610,615.3648,455.3137,425.3034

126	Butenyl group-Rd	59.41	$C_{52}H_{86}O_{19}$	1059.5722	-4.0	[M-H+HCOO]$^-$	1013.5604,945.5368,927.5260,783.4842,765.4734, 621.4321,
127	Ginsenoside Rg_3	59.69	$C_{42}H_{72}O_{13}$	829.5012	5.7	[M-H+HCOO]$^-$	783.4806,621.4456,459.3907,375.2970,
128	20(S)-6″-O-acetylginsenoside Rg_3	61.83	$C_{44}H_{74}O_{14}$	871.5101	2.8	[M-H+HCOO]$^-$	783.4993,765.4890,621.4451,459.3924
129	Veraguensin	61.93	$C_{22}H_{28}O_5$	373.1956	6.7	[M+H]$^-$	235.1325,217.1221,191.1064,167.0701,151.0752
130	Poricoic acid HM	62.82	$C_{32}H_{50}O_5$	513.3645	-1.4	[M-H]$^-$	469.3403,453.3453,441.3087
131	Magnosalicin	63.07	$C_{24}H_{32}O_7$	433.2165	5.3	[M+H]$^-$	415.2113,401.1956,369.3148,265.1432,247.1327,181.0858
132	Poricoic acid GM	63.37	$C_{31}H_{48}O_5$	499.3486	-2.0	[M-H]$^-$	469.3296,455.3594,397.3180,353.2547
133	24(R)-pseudoginsenoside RT_5/isomer	64.04	$C_{33}H_{58}O_{14}$	723.3864	5.6	[M-H+HCOO]$^-$	677.3842,397.1423
134	16-Deoxyporicoic acid B	64.27	$C_{30}H_{44}O_4$	467.3226	-1.7	[M-H]$^-$	452.2993,437.2757,423.2597,407.3010,313.2424
135	(-)-cadala-1,4,9-triene	64.98	$C_{15}H_{22}$	203.1736	9.9	[M+H]$^-$	175.1479,161.1323,147.1166
136	Poriacosone B	64.99	$C_{30}H_{46}O_5$	485.3338	-0.2	[M-H]$^-$	485.2895,469.2580,439.3280,386.1708,337.2220
137	24(R)-pseudoginsenoside RT_5/isomer	65.25	$C_{33}H_{58}O_{14}$	723.3845	2.8	[M-H+HCOO]$^-$	677.3843,397.1417
138	3β,16-α-Dihydroxylanosta7,9(11),24-trien-21-oic acid	65.91	$C_{30}H_{46}O_4$	469.3387	-0.6	[M-H]$^-$	469.2580,451.3280,423.3328,407.3376,337.2583
139	6α-Hydroxydehydropachymic acid	66.1	$C_{33}H_{50}O_6$	541.3604	0.6	[M-H]$^-$	523.3494,497.3338,481.3387,445.2669
140	Longicyclene	66.24	$C_{15}H_{24}$	205.1864	-4.2	[M+H]$^-$	163.1480,149.1324,135.1167,121.1010
141	2S-(2,6-Dimethoxy-4-propenyl-phenoxy)-1 -(3,4,5-trimethoxy-phenyl)-propane-1-one	66.3	$C_{23}H_{28}O_7$	417.1848	4.6	[M+H]$^-$	385.1643,357.1693,249.1120,221.1171,193.0857,165.0909
142	16α-Hydroxytrametenolic acid	66.36	$C_{30}H_{48}O_4$	471.3542	-1.1	[M-H]$^-$	453.3436,425.3482,409.3167,407.3375,337.2581
143	Poricoic acid B	66.79	$C_{30}H_{44}O_5$	483.3172	-2.3	[M-H]$^-$	465.3072,439.3275,421.3168,409.2802
144	Poricoic acid E	67.49	$C_{32}H_{48}O_6$	527.3444	-0.2	[M-H]$^-$	509.3339,481.3020,451.3293,409.2819
145	Dehydrotumulosic acid	67.64	$C_{31}H_{48}O_4$	483.3169	-2.9	[M-H]$^-$	465.3439,439.3644,409.3533
146	1,3-Dimethoxy-2- [1-methyl-2-(3,4,5-trimethoxyphenyl)- ethoxy]-5-(1-	67.73	$C_{23}H_{30}O_6$	403.2045	2.2	[M-H]$^-$	235.1327,209.1169,195.1014,181.0857

	propenyl-1-yl)-benzene						
147	Poricoic acid G	67.78	$C_{30}H_{46}O_5$	485.3369	6.2	$[M-H]^-$	485.2895,441.3799,423.3327,353.2534,337.2583
148	Dehydroeburicoic acid	67.84	$C_{31}H_{47}O_3$	466.3547	6.0	$[M-H]^-$	423.3242,407.2930,371.2207,353.2102
149	Tumulosic acid	67.92	$C_{31}H_{50}O_4$	485.3673	-7.6	$[M-H]^-$	437.3416,423.3364,337.2564
150	Poricoic acid A	68.1	$C_{31}H_{46}O_5$	497.334	0.2	$[M-H]^-$	479.3234,453.3437,423.2978,381.3230,274.1778
151	Calacone	68.11	$C_{15}H_{24}O$	221.1824	1.4	$[M+H]^-$	203.1792,160.1200,147.1168,133.1011
152	25-Hydroxypachymic acid	68.15	$C_{33}H_{52}O_6$	453.3746	-2.2	$[M-H]^-$	525.3669,481.3389,467.3595,465.3439
153	26-Hydroxyporicoic acid DM	68.46	$C_{32}H_{48}O_7$	543.3363	8.0	$[M-H]^-$	543.9825,525.3645,465.3452,415.2926
154	3-O-Acetyl-16α-hydroxytrametenolic acid	68.52	$C_{32}H_{50}O_5$	513.3686	6.6	$[M-H]^-$	495.3139,467.3594,451.3278,417.2858,355.2326,315.2583
155	Dehydropachymic acid	68.79	$C_{33}H_{50}O_5$	525.3634	-3.4	$[M-H]^-$	509.3293,465.3449,447.3328,355.2340,337.2232
156	Poricoic acid AE	69.02	$C_{33}H_{50}O_5$	525.3649	-5.6	$[M-H]^-$	509.3297,465.3436,447.3328,355.2340,337.2232
157	Polyporenic acid C	69.3	$C_{31}H_{46}O_4$	483.3364	-8.5	$[M-H]^-$	481.3061,463.3488,437.3481,421.3167,403.3057,271.1738
158	3-epidehydrotumulosic acid	69.5	$C_{31}H_{48}O_4$	483.3499	-4.1	$[M-H]^-$	421.3157,391.2278
159	3β-hydroxylanosta-8,24-dien -21-oic acid	70.01	$C_{30}H_{48}O_3$	455.3561	-6.8	$[M-H]^-$	455.1752,297.2416,279.2316
160	25-hydroxy-3-epitumulosic acid	73.98	$C_{31}H_{50}O_4$	485.3657	-4.3	$[M-H]^-$	485.2791,423.3316,337.2517

Table S2 The typical filtered ion, related parameters and the compound identification

No.	Identification	Filtered ion	CK vs CG			MK vs MG		
			VIP	P	FC	VIP	P	FC
1	Polygalaxanthone VI	493.1367#,317.064	1.523	0.000011	1261856	/	/	/
2	Polygalaxanthone III	567.1376#,345.0594,315.0491	1.722	0.00182	953273	/	/	/
3	Ginsenoside Rg_1	845.4909#,799.4956,637.4409,475.3821	2.147	0.00017	993	/	/	/
4	Ginsenoside Re	991.5489#,783.4943,637.4467,475.3864	1.723	0.01983	200530	/	/	/
5	Polygalasaponin XXIX	1397.6189#,455.3138,425.30354	1.523	0.00075	146463	/	/	/
6	Desacylsenegasaponin B	1265.5851#,1235.5604,455.3139,425.3035	1.755	0.01113	117574	1.696	0.0322	114137
7	Tenuifoliose O	1367.4108#,455.3167,425.3062	1.514	0.00143	161935	/	/	/
8	Polygalasaponin XXII	1235.5778#,455.3136,425.3033	1.827	0.00499	684882	/	/	/
9	Polygalasaponin XXVIII	1103.5304#,1073.5092,455.3137,425.3034	1.525	0.022886	401914	/	/	/
10	Ginsenoside Rb1	1153.6083#,945.536,783.4845,621.433,459.3814	1.632	0.00013	1014187	1.752	0.01455	250667
11	Ginsenoside Ro	955.4928#,793.4369,631.3758	1.521	0.01016	77063	/	/	/
12	Ginsenoside Rc	1123.5941#,945.5347,783.4834,621.4321,459.3809	1.647	0.00644	51631	1.756	0.0297	434656
13	Ginsenoside Ra_1	1255.6387#,1077.5786,783.4869,459.3911	1.575	0.03339	45488	1.599	0.02493	22631
14	Ginsenoside Rb_2	1123.5916#,945.5379,783.4856,621.4367,459.3897	2.672	0.00019	729516	1.808	0.0403	158564
15	Ginsenoside Rd	991.5496#,459.3908	1.687	0.01649	82118	1.687	0.0897	209284
16	16α-hydroxytrametenolic acid	471.3497#,409.3156,337.2567	2.448	0.00074	184481	/	/	/
17	Poricoic acid B	483.3125#,465.3675,409.2798	2.049	0.00094	510906	1.687	0.029042	209175
18	Dehydrotumulosic acid	483.3496#,421.2674,337.25687	2.170	0.00116	464072	/	/	/
19	Tumulosic acid	485.3637#,437.3489,337.2185	2.264	0.0002	17088	1.755	0.082027	332532
20	Poricoic acid A	497.3287#,409.2786,381.3687	2.050	0.00098	118248	1.921	0.01078	279313
21	Polyporenic acid C	481.3305#,437.2965	2.035	0.00059	132907	/	/	/
22	Poricoic acid B isomer	483.3123#,439.5491,255.2367	/	/	/	1.846	0.027991	415380
23	3-epidehydrotumulosic acid	483.3511#,421.3157,391.3581	1.647	0.01506	186645	/	/	/

24	3β-hydroxylanosta-8,24-dien -21-oic acid	455.3566#,279.2346	1.675	0.00338	11537	/	/	/
25	Poricoic acid A isomer	497.3315#,437.3515	1.674	0.00111	767607	/	/	/
26	25-hydroxy-3-epitumulosic acid	485.3616#,423.3265,337.2657	/	/	/	1.816	0.028063	812235
27	Hydrated ginsenoside Rb_1 (+$2H_2O$)	1143.6178#,783.4861	2.072	0.0001	674894	/	/	/
28	Oxidated ginsenoside Rb1	1123.5879#,478.9265	2.402	0.000061	553404	/	/	/
29	Ginsenoside F_2	829.4933#,459.3731	1.602	0.04796	21796	1.657	0.012941	641382
30	Dehydrotumulosic acid hydroxylation	499.3442#,437.3465,421.3068	1.826	0.00188	285709	/	/	/
31	Tumulosic acid hydroxylation + Desaturation	499.3412#,437.3395,421.3419	1.614	0.00623	4078703	/	/	/
32	Tumulosic acid hydroxylation	501.359#,373.2961	1.549	0.00113	1285076	/	/	/
33	Dehydrotumulosic acid hydroxylation	499.3412#,421.3085	1.602	0.00027	952264	/	/	/
34	Hydrated Pachymic acid	527.3885#,465.3347	/	/	/	1.625	0.029665	1205430
35	Poricoic acid G dehydration + Glycine conjugation	524.3406#,464.3751	/	/	/	1.605	0.01003	904245
36	Dehydrotumulosic acid demethylation	469.3342#,409.3364,337.2647	1.866	0.00173	112797	/	/	/
37	16α-hydroxytrametenolic acid hydroxylation	487.3453#,469.3647	1.633	0.00033	456381	/	/	/
38	Dehydrotumulosic acid Desaturation	481.3349#,437.3691	1.512	0.0031	338921	/	/	/
39	Poricoic acid B Glycine conjugation	540.3297#,409.3693	1.647	0.00065	68752	/	/	/
40	Tumulosic acid demethylation	471.3545#,409.611,375.2657,	1.605	0.02096	57006	1.825	0.079238	2778182
41	Poricoic acid G dehydration + deoxygenation+Desaturation+Glycine conjugation	494.3674#,295.6174	/	/	/	1.508	0.015229	41317
42	Poricoic acid G Oxidation+Glycine conjugation	540.3269#,481.2657	/	/	/	1.829	0.01988	372061
43	Oxidated pachymic acid	525.3539#,481.3564,465.6214	/	/	/	1.792	0.00297	701676
44	Dehydrotumulosic acetylation	525.3567#,465.3664	/	/	/	1.792	0.029327	701676
45	Poricoic acid G deoxidation（-2O）	453.3361#,323.2647	1.563	0.00024	3743413	1.734	0.010993	4701453

[#]: The ions in red whose VIP values are in “VIP” columns

Table S3 The compound library of KXS

No.	Compound	Formula	Measured mass (m/z)	Origin
1	Lactic acid	$C_3H_6O_3$	89.0239	Polygala
2	Malonic acid	$C_3H_4O_4$	103.0032	Polygala
3	Glyceric acid	$C_3H_6O_4$	105.0188	Polygala
4	Fumaric acid	$C_4H_4O_4$	115.0032	Polygala
5	Succinic acid	$C_4H_6O_4$	117.0188	Polygala
6	Glutaric acid	$C_5H_8O_4$	131.0345	Polygala
7	Malic acid	$C_4H_6O_5$	133.0137	Polygala
8	Salicylic Acid	$C_7H_6O_3$	137.0251	Ginseng
9	Adipic acid	$C_6H_{10}O_4$	145.0501	Polygala
10	Hexanedioic acid	$C_6H_{10}O_4$	145.0501	Polygala
11	Xylose	$C_5H_{10}O_5$	149.045	Polygala
12	Arabinose	$C_5H_{10}O_5$	149.045	Polygala
13	Apiose	$C_5H_{10}O_5$	149.045	Polygala
14	Fucose	$C_6H_{12}O_5$	163.0607	Polygala
15	Rhamnose	$C_6H_{12}O_5$	163.0607	Polygala
16	Arabic acid	$C_5H_{10}O_6$	165.0399	Polygala
17	Glucose	$C_6H_{12}O_6$	179.0556	Polygala
18	Fructose	$C_6H_{12}O_6$	179.0556	Polygala
19	Galactose	$C_6H_{12}O_6$	179.0556	Polygala
20	Citric acid	$C_6H_8O_7$	191.0192	Polygala
21	Citric Acid	$C_6H_8O_7$	191.0194	Ginseng
22	Quinic acid	$C_7H_{12}O_6$	191.0556	Polygala
23	Ferulic acid	$C_{10}H_{10}O_4$	193.0501	Polygala
24	Gluconic acid	$C_6H_{12}O_7$	195.0505	Polygala
25	Heptopyranose	$C_7H_{14}O_7$	209.0662	Polygala
26	Vanillactic acid	$C_{10}H_{12}O_5$	211.0607	Polygala
27	Sinapinic acid	$C_{11}H_{12}O_5$	223.0607	Polygala
28	Euxanthone	$C_{13}H_8O_4$	227.0345	Polygala
29	1,3-Dihydroxyxanthone	$C_{13}H_8O_4$	227.0345	Polygala
30	1,7-Dihydroxyxanthone	$C_{13}H_8O_4$	227.0345	Polygala
31	7-Hydroxy-1-methoxyxanthone	$C_{14}H_{10}O_4$	241.0501	Polygala
32	1,3-Dihydroxy-2-methylxanthone	$C_{14}H_{10}O_4$	241.0501	Polygala
33	1,2,8-Trihydroxyxanthone	$C_{13}H_8O_5$	243.0294	Polygala
34	1,3,6-Trihydroxyxanthone	$C_{13}H_8O_5$	243.0294	Polygala
35	Gentitein	$C_{13}H_8O_5$	243.0294	Polygala
36	1-Hydroxy-2,3-methylenedioxyxanthone	$C_{14}H_8O_5$	255.0294	Polygala
37	1,7-Dimethoxyxanthone	$C_{15}H_{12}O_4$	255.0658	Polygala
38	7-Hydroxy-1,2-dimethoxyxanthone	$C_{14}H_{10}O_5$	257.045	Polygala
39	7-Hydroxy-1,3-dimethoxyxanthone	$C_{14}H_{10}O_5$	257.045	Polygala
40	1,3-Dihydroxy-2-methoxyxanthone	$C_{14}H_{10}O_5$	257.045	Polygala
41	1,3-Dihydroxy-7-methoxyxanthone	$C_{14}H_{10}O_5$	257.045	Polygala

42	1,7-Dihydroxy-3-methoxyxanthone	$C_{14}H_{10}O_5$	257.045	Polygala
43	1,7-Dihydroxy-4-methxoyxanthone	$C_{14}H_{10}O_5$	257.045	Polygala
44	2,7-Hydroxy-1-dimethoxyxanthone	$C_{14}H_{10}O_5$	257.045	Polygala
45	Alpestriose A	$C_{15}H_{16}O_4$	259.0971	Polygala
46	1-Methoxy-2,3-methylenedioxyxanthone	$C_{15}H_{10}O_5$	269.045	Polygala
47	1,7-Dihydroxy-2,3-methylenedioxyxanthone	$C_{14}H_8O_6$	271.0243	Polygala
48	1,4-Dihydroxy-6,7-methylenedioxyxanthone	$C_{14}H_8O_6$	271.0243	Polygala
49	4,7-Dihydroxy-2,3-methylenedioxyxanthone	$C_{14}H_8O_6$	271.0243	Polygala
50	1-Hydroxy-2,3-dimethoxyxanthone	$C_{15}H_{12}O_5$	271.0607	Polygala
51	1-Hydroxy-2,4-dimethoxyxanthone	$C_{15}H_{12}O_5$	271.0607	Polygala
52	1-Hydroxy-3,7-dimethoxyxanthone	$C_{15}H_{12}O_5$	271.0607	Polygala
53	2-Hydroxy-3,4-dimethoxyxanthone	$C_{15}H_{12}O_5$	271.0607	Polygala
54	3-Hydroxy-1,2-dimethoxyxanthone	$C_{15}H_{12}O_5$	271.0607	Polygala
55	3-Hydroxy-1,4-dimethoxyxanthone	$C_{15}H_{12}O_5$	271.0607	Polygala
56	3-Hydroxy-2,8-dimethoxyxanthone	$C_{15}H_{12}O_5$	271.0607	Polygala
57	1,3,7-Trihydroxy-2-methoxyxanthone	$C_{14}H_{10}O_6$	273.0399	Polygala
58	1,2,7-Trihydroxy-3-methoxyxanthone	$C_{14}H_{10}O_6$	273.0399	Polygala
59	1,4,7-Trihydroxy-3-methoxyxanthone	$C_{14}H_{10}O_6$	273.0399	Polygala
60	1-Hydroxy-5-methoxy-2,3-methylenedioxyxanthone	$C_{15}H_{10}O_6$	285.0399	Polygala
61	6-Hydroxy-1-methoxy-2,3-methylenedioxyxanthone	$C_{15}H_{10}O_6$	285.0399	Polygala
62	7-Hydroxy-1-methoxy-2,3-methylenedioxyxanthone	$C_{15}H_{10}O_6$	285.0399	Polygala
63	1,2,3-Trimethoxyxanthone	$C_{16}H_{14}O_5$	285.0763	Polygala
64	1,6,8-Trihydroxy-2,3-methylenedioxyxanthone	$C_{14}H_8O_7$	287.0192	Polygala
65	1,5-Dihydroxy-2,3-dimethoxyxanthone	$C_{15}H_{12}O_6$	287.0556	Polygala
66	1,6-Dihydroxy-3,7-dimethoxyxanthone	$C_{15}H_{12}O_6$	287.0556	Polygala
67	1,7-Dihydroxy-2,3-dimethoxyxanthone	$C_{15}H_{12}O_6$	287.0556	Polygala
68	1,7-Dihydroxy-3,4-dimethoxyxanthone	$C_{15}H_{12}O_6$	287.0556	Polygala
69	1,8-Dihydroxy-2,7-dimethoxyxanthone	$C_{15}H_{12}O_6$	287.0556	Polygala
70	2,6-Dihydroxy-1,5-dimethoxyxanthone	$C_{15}H_{12}O_6$	287.0556	Polygala
71	3,7-Dihydroxy-1, 2-dimethoxyxanthone	$C_{15}H_{12}O_6$	287.0556	Polygala
72	3,8-Dihydroxy-1,2-dimethoxyxanthone	$C_{15}H_{12}O_6$	287.0556	Polygala
73	1-Hydroxy-2,3,6,7-bimethylenedioxyxanthone	$C_{15}H_8O_7$	299.0192	Polygala
74	1,4-Dimethoxy-2,3-methylenedioxyxanthone	$C_{16}H_{12}O_6$	299.0556	Polygala
75	1,7-Dimethoxy-2,3-methylenedioxyxanthone	$C_{16}H_{12}O_6$	299.0556	Polygala
76	1,8-Dimethoxy-2,3-methylenedioxyxanthone	$C_{16}H_{12}O_6$	299.0556	Polygala
77	1-Hydroxy-3,6,7-trimethoxyxanthone	$C_{16}H_{14}O_6$	301.0712	Polygala
78	2-Hydroxy-1,6,7-trimethoxyxanthone	$C_{16}H_{14}O_6$	301.0712	Polygala
79	3-Hydroxy-1,2,7-trimethoxyxanthone	$C_{16}H_{14}O_6$	301.0712	Polygala
80	Onjixanthone I	$C_{16}H_{14}O_6$	301.0712	Polygala

81	Onjixanthone II	$C_{15}H_{12}O_7$	303.0505	Polygala
82	1,3,7-Trihydroxy-2,6-dimethoxyxanthone	$C_{15}H_{12}O_7$	303.0505	Polygala
83	1,3,8-Trihydroxy-2,6-dimethoxyxanthone	$C_{15}H_{12}O_7$	303.0505	Polygala
84	1,6,7-Trihydroxoy-2,3-dimethoxyxanthone	$C_{15}H_{12}O_7$	303.0505	Polygala
85	1-Methoxy-2,3,6,7-bimethylenedioxyxanthone	$C_{16}H_{10}O_7$	313.0349	Polygala
86	2-Hydroxy-1,3-dimethoxy-7,8-methylenedioxyxanthone	$C_{16}H_{12}O_7$	315.0505	Polygala
87	1,2,3,7-Tetramethoxyxanthone	$C_{17}H_{16}O_6$	315.0869	Polygala
88	1,3,6,7-Tetramethoxyxanthone	$C_{17}H_{16}O_6$	315.0869	Polygala
89	1,6,8-Trihydroxy-7-methoxy-2,3-methylenedioxyxanthone	$C_{15}H_{10}O_8$	317.0298	Polygala
90	1,3-Dihydroxy-2,4,7-trimethoxyxanthone	$C_{16}H_{14}O_7$	317.0662	Polygala
91	1,3-Dihydroxy-5,6,7-trimethoxyxanthone	$C_{16}H_{14}O_7$	317.0662	Polygala
92	1,6-Dihydroxy-3,5,7- trimethoxyxanthone	$C_{16}H_{14}O_7$	317.0662	Polygala
93	1,7-Dihydroxy-2,3,4- trimethoxyxanthone	$C_{16}H_{14}O_7$	317.0662	Polygala
94	1,7-Dihydroxy-3,5,6-trimethoxyxanthone	$C_{16}H_{14}O_7$	317.0662	Polygala
95	3,6-Dihydroxy-1,2,7-trimethoxyxanthone	$C_{16}H_{14}O_7$	317.0662	Polygala
96	3,6-Dihydroxy-1,2,8-trimethoxyxanthone	$C_{16}H_{14}O_7$	317.0662	Polygala
97	3,8-Dihydroxy-1,2,6-trimethoxyxanthone	$C_{16}H_{14}O_7$	317.0662	Polygala
98	6,8-Dihydroxy-1,2,3-trimethoxyxanthone	$C_{16}H_{14}O_7$	317.0662	Polygala
99	6,8-Dihydroxy-1,2,4-trimethoxyxanthone	$C_{16}H_{14}O_7$	317.0662	Polygala
100	1,3,6,8-Tetrahydroxy-2,7-dimethoxyxanthone	$C_{15}H_{12}O_8$	319.0454	Polygala
101	Polygalaxanthone A	$C_{17}H_{14}O_7$	329.0662	Polygala
102	1,2,3-Trimethoxy-7,8-methylenedioxyxanthone	$C_{17}H_{14}O_7$	329.0662	Polygala
103	1,6-Dihydroxy-7,8-dimethoxy-2,3-methylenedioxyxanthone	$C_{16}H_{12}O_8$	331.0454	Polygala
104	6-Hydroxy-1,2,3,7-tetramethoxyxanthone	$C_{17}H_{16}O_7$	331.0818	Polygala
105	7-Hydroxy-1,2,3,4-tetramethoxyxanthone	$C_{17}H_{16}O_7$	331.0818	Polygala
106	1,3,6-Trihydroxy-2,7,8-trimethoxyxanthone	$C_{16}H_{14}O_8$	333.0611	Polygala
107	Sucrose	$C_{12}H_{22}O_{11}$	341.1084	Polygala
108	cis-Coniferin	$C_{16}H_{22}O_8$	341.1237	Polygala
109	Polygalaxanthone B	$C_{18}H_{18}O_7$	345.0975	Polygala
110	1,2,3,6,7-Pentamethoxyxanthone	$C_{18}H_{18}O_7$	345.0975	Polygala
111	1,2,3,7,8-Pentamethoxyxanthone	$C_{18}H_{18}O_7$	345.0975	Polygala
112	1,3-Dihydroxy-2,5,6,7-tetramethoxyxanthone	$C_{17}H_{16}O_8$	347.0767	Polygala
113	3,6-Dihydroxy-1,2,7,8-tetramethoxyxanthone	$C_{17}H_{16}O_8$	347.0767	Polygala
114	pregn-7-ene-2β,3α,15α,20(S)-tetro	$C_{21}H_{34}O_4$	349.23843	Poriacocos
115	3-Hydroxy-1,2,5,6,7-pentamethoxyxanthone	$C_{18}H_{18}O_8$	361.0924	Polygala
116	Syringin	$C_{17}H_{24}O_9$	371.1342	Polygala
117	1,2,3,4,6,7-Hexamethoxyxanthone	$C_{19}H_{20}O_8$	375.108	Polygala
118	Tenuifoliside D	$C_{18}H_{24}O_9$	383.1342	Polygala
119	Wubangziside B	$C_{19}H_{18}O_9$	389.0873	Polygala

120	ergosta-4,22-dien-3-one	$C_{28}H_{44}O$	395.33193	Poriacocos
121	ergosta-5,7,22-trien-3β-ol	$C_{28}H_{44}O$	395.331939	Poriacocos
122	ergosta-7,22-dien-3-one	$C_{28}H_{44}O$	395.331939	Poriacocos
123	ergosta-7,22-dien-3β-ol	$C_{28}H_{46}O$	397.3475	Poriacocos
124	ergosta-7-en-3β-ol	$C_{28}H_{48}O$	399.36323	Poriacocos
125	Wattersiixanthone B	$C_{20}H_{20}O_9$	403.1029	Polygala
126	Neolancerin	$C_{19}H_{18}O_{10}$	405.0822	Polygala
127	Lancerin	$C_{19}H_{18}O_{10}$	405.0822	Polygala
128	6,9-epoxy-ergosta-7,22-dien-3β-ol	$C_{28}H_{44}O_2$	411.3268	Poriacocos
129	ergosta-5,6-epoxy-7,22-dien-3-ol	$C_{28}H_{44}O_2$	411.326853	Poriacocos
130	stigmasterol	$C_{29}H_{48}O$	411.36323	Poriacocos
131	β-sitosterol	$C_{29}H_{50}O$	413.378889	Poriacocos
132	1-Glucosyloxy-2,3-methylenedioxyxanthone	$C_{20}H_{18}O_{10}$	417.0822	Polygala
133	1-Glucosyloxy-2-hydroxy-3-methoxyxanthon e	$C_{20}H_{20}O_{10}$	419.0978	Polygala
134	Mangiferin	$C_{19}H_{18}O_{11}$	421.0771	Polygala
135	Isomangiferin	$C_{19}H_{18}O_{11}$	421.0771	Polygala
136	Guazijinxanthone	$C_{24}H_{24}O_7$	423.1444	Polygala
137	9,11-dehydroergosta peroxide	$C_{28}H_{42}O_3$	425.30611	Poriacocos
138	lupeol	$C_{30}H_{50}O$"	425.3788	Poriacocos
139	3β,5α,9α-trihydroxyergosta-7,22-dien-6-one	$C_{28}H_{44}O_3$	427.3217	Poriacocos
140	ergosta-6,22-dien-5α,8α-epidioxy-3-ol	$C_{28}H_{44}O_3$	427.3217	Poriacocos
141	3β,5α-dihydroxyergosta-7,22-dien-6-one	$C_{28}H_{44}O_3$	427.32176	Poriacocos
142	Polygalatenoside A	$C_{19}H_{26}O_{11}$	429.1397	Polygala
143	Polygalatenoside B	$C_{19}H_{26}O_{11}$	429.1397	Polygala
144	Polygalatenoside C	$C_{19}H_{26}O_{11}$	429.1397	Polygala
145	cerevisterol	$C_{28}H_{46}O_3$	429.337418	Poriacocos
146	Tricornoside E	$C_{20}H_{20}O_{11}$	435.0928	Polygala
147	4-C-β-Glucopyranosyl-1,3,6-trihydroxy-7-me thoxyxanthone	$C_{20}H_{20}O_{11}$	435.0928	Polygala
148	7-O-methoxymangiferin	$C_{20}H_{20}O_{11}$	435.0928	Polygala
149	6-O-benzoylsucrose	$C_{19}H_{26}O_{12}$	445.1346	Polygala
150	Canthoside A	$C_{19}H_{26}O_{12}$	445.1346	Polygala
151	Sibiricaxanthone C	$C_{21}H_{22}O_{11}$	449.1084	Polygala
152	Polyhongkongenoside B	$C_{21}H_{22}O_{11}$	449.1084	Polygala
153	porilactone B	$C_{30}H_{44}O_3$	451.3217	Poriacocos
154	porilactone A	$C_{30}H_{44}O_3$	451.32176	Poriacocos
155	Dehydrotrametenolic acid	$C_{30}H_{46}O_3$	453.3351	Poriacocos
156	3β-Hydroxylanosta-7,9(11),24-trien-21-oic acid	$C_{30}H_{46}O_3$	453.3372	Poriacocos
157	Pinicolic acid	$C_{30}H_{46}O_3$	453.3669	Poriacocos
158	oleanolic acid	$C_{30}H_{48}O_3$	455.353	Poriacocos
159	trametenolic acid	$C_{30}H_{48}O_3$	455.353	Poriacocos
160	ursolic acid	$C_{30}H_{48}O_3$	455.35306	Poriacocos

161	Sibiricose A3	$C_{19}H_{26}O_{13}$	461.1295	Polygala
162	daedaleanic acid E	$C_{30}H_{42}O_4$	465.301033	Poriacocos
163	3−Oxo−16α−hydroxylanosta−7,9(11),24−trien−21−oic acid	$C_{30}H_{44}O_4$	467.3158	Poriacocos
164	16-deoxyporicoic acid B	$C_{30}H_{44}O_4$	467.31668	Poriacocos
165	dehydroeburiconic acid	$C_{31}H_{48}O_3$	467.35306	Poriacocos
166	β-amyrin acetate	$C_{32}H_{52}O_2$"	467.3894	Poriacocos
167	α-amyrin acetate	$C_{32}H_{52}O_2$	467.38945	Poriacocos
168	16α-Hydroxy-3-oxolanosta-8,24-dien-21-oic acid	$C_{30}H_{46}O_4$	469.3304	Poriacocos
169	3α,16β−Dihydroxylanosta−7,9(11),24−trien−21−oic acid	$C_{30}H_{46}O_4$	469.3316	Poriacocos
170	Poriacosones B	$C_{30}H_{46}O_4$	469.33194	Poriacocos
171	16α-hydroxydehydrotrametenolic acid	$C_{30}H_{46}O_4$"	469.3323	Poriacocos
172	3α,16β−Dihydroxylanosta−7,9(11),24−trien−21−oic acid	$C_{30}H_{46}O_4$	469.3328	Poriacocos
173	3α,16β−Dihydroxylanosta-7,9(11),24−trien−21−oic acid	$C_{30}H_{46}O_4$	469.3332	Poriacocos
174	Pinicolic acid E	$C_{30}H_{46}O_4$	469.3332	Poriacocos
175	eburicoic acid	$C_{31}H_{50}O_3$	469.73	Poriacocos
176	Hederagenin	$C_{30}H_{48}O_4$	471.3475	Polygala
177	16α-hydroxytrametenolic acid	$C_{30}H_{48}O_4$	471.34798	Poriacocos
178	Polygalatenoside D	$C_{20}H_{28}O_{13}$	475.1452	Polygala
179	daedaleanic acid F	$C_{31}H_{42}O_4$	477.30103	Poriacocos
180	3−Oxo−16α−hydroxylanosta−7,9(11),24−trien−21−oic acid	$C_{31}H_{44}O_4$	479.316	Poriacocos
181	daedaleanic acid D	$C_{31}H_{44}O_4$	479.31668	Poriacocos
182	poricoic acid C	$C_{31}H_{46}O_4$	481.33233	Poriacocos
183	polyporenic acid C	$C_{31}H_{46}O_4$	481.7	Poriacocos
184	6,16α-Dihydroxydehydrotrametenonic acid	$C_{30}H_{44}O_5$	483.3101	Poriacocos
185	3,24Dioxo16αhydroxylanosta7,9(11) dien21oic acid	$C_{30}H_{44}O_5$	483.3112	Poriacocos
186	16-Hydroxy-3,24-dioxolanosta-7,9(11)-dien-21-oic acid	$C_{30}H_{44}O_5$	483.31131	Poriacocos
187	poricoic acid B	$C_{30}H_{44}O_5$	483.31159	Poriacocos
188	15α-Hydroxyeburiconic acid	$C_{31}H_{48}O_4$	483.3468	Poriacocos
189	3-epi-dehydrotumulosic acid	$C_{31}H_{48}O_4$"	483.3479	Poriacocos
190	dehydrotumulosic acid	$C_{31}H_{48}O_4$	483.34798	Poriacocos
191	16α,27-Dihydroxydehydrotrametenoic acid	$C_{30}H_{46}O_5$	485.3258	Poriacocos
192	Poriacosone B	$C_{30}H_{46}O_5$	485.3265	Poriacocos
193	Poriacosone A	$C_{30}H_{46}O_5$	485.327	Poriacocos
194	3-O-Acetyl-16α-hydroxytrametenolic acid	$C_{30}H_{46}O_5$	485.3273	Poriacocos
195	Tumulosic acid	$C_{31}H_{50}O_4$	485.3636	Poriacocos
196	16α-hydroxyeburicoic acid	$C_{31}H_{50}O_4$	485.36363	Poriacocos

197	25-hydroxy-3-epitumulosic acid	$C_{31}H_{50}O_4$	485.36363	Poriacocos
198	Hispindic acid B	$C_{31}H_{50}O_4$	485.3643	Poriacocos
199	poricoic acid G	$C_{30}H_{46}O_5$	485.68	Poriacocos
200	Polygalic acid	$C_{29}H_{44}O_6$	487.306	Polygala
201	Daedaleanic acid B	$C_{30}H_{48}O_5$	487.3415	Poriacocos
202	3α,16α,26-Trihydroxylanosta-8,24dien-21-oic acid	$C_{30}H_{48}O_5$	487.34299	Poriacocos
203	Polygalaxanthone VI	$C_{23}H_{26}O_{12}$	493.1346	Polygala
204	3β−Acetoxylanosta−7,9(11),24−trien−21−oic acid	$C_{32}H_{48}O_4$	495.3462	Poriacocos
205	poricoic acid CM	$C_{32}H_{48}O_4$	495.34798	Poriacocos
206	6,16α-Dihydroxydehydroeburiconic acid	$C_{31}H_{46}O_5$	497.3263	Poriacocos
207	25-hydroxypolyporenic acid C	$C_{31}H_{46}O_5$	497.32724	Poriacocos
208	29-hydroxypolyporenic acid C	$C_{31}H_{46}O_5$	497.32724	Poriacocos
209	poricoic acid A	$C_{31}H_{46}O_5$	497.32724	Poriacocos
210	poricoic acid BM	$C_{31}H_{46}O_5$	497.32724	Poriacocos
211	6,7-dehydroporicoic acid H	$C_{31}H_{46}O_5$	497.327247	Poriacocos
212	16α,25-Dihydroxydehydroeburiconic acid	$C_{31}H_{46}O_5$	497.3273	Poriacocos
213	3α−Acetoxylanosta−8,24−dien−21−oic acid	$C_{32}H_{46}O_4$	497.3622	Poriacocos
214	3-O-formyleburicoic acid	$C_{32}H_{50}O_4$	497.36363	Poriacocos
215	oleanolic acid 3-O-acetate	$C_{32}H_{50}O_4$	497.36363	Poriacocos
216	6α-hydroxypolyporenic acid C	$C_{31}H_{46}O_5$	497.69	Poriacocos
217	poricoic acid E	$C_{30}H_{44}O_6$	499.3065	Poriacocos
218	16α,25-Dihydroxyeburiconic acid	$C_{31}H_{48}O_5$	499.3408	Poriacocos
219	25-Hydroxy-3-epidehydrotumulosic acid	$C_{31}H_{48}O_5$	499.3408	Poriacocos
220	16α,29-Dihydroxyeburiconic acid	$C_{31}H_{48}O_5$	499.3412	Poriacocos
221	3β,16α−Dihydroxy−7−oxolanosta−8,24−dien −21−oic acid	$C_{31}H_{48}O_5$	499.3425	Poriacocos
222	poricoic acid GM	$C_{31}H_{48}O_5$	499.3428	Poriacocos
223	15α-hydroxydehydrotumulosic acid	$C_{31}H_{48}O_5$	499.34289	Poriacocos
224	6α-hydroxydehydrotumulosic acid	$C_{31}H_{48}O_5$	499.342897	Poriacocos
225	poricoic acid H	$C_{31}H_{48}O_5$	499.342897	Poriacocos
226	26-hydroxyporicoic acid G	$C_{30}H_{46}O_6$	501.32216	Poriacocos
227	pinicolic acid F	$C_{30}H_{46}O_6$	501.32216	Poriacocos
228	3β,16α-Dihydroxy-24-hydroxymethyllanosta-7,9(11)-dien-21-oic acid	$C_{31}H_{50}O_5$	501.3564	Poriacocos
229	25α-Hydroxytumulosic acid	$C_{31}H_{50}O_5$	501.3572	Poriacocos
230	16α,25-dihydroxyeburicoic acid	$C_{31}H_{50}O_5$	501.35854	Poriacocos
231	Maltotriose	$C_{18}H_{32}O_{16}$	503.1612	Polygala
232	Polygalatenoside E	$C_{22}H_{32}O_{13}$	503.1765	Polygala
233	Polygalacic acid	$C_{30}H_{48}O_6$	503.3373	Polygala
234	Poricoic acid CE	$C_{33}H_{50}O_4$	509.361	Poriacocos
235	3-O-Acetyl-16α-hydroxydehydrotrametenolic acid	$C_{32}H_{48}O_5$	511.3423	Poriacocos

236	3β-Hydroxy-16α-acetoxylanosta-7,9(11),24-trien-21-oic acid	$C_{32}H_{48}O_5$	511.3426	Poriacocos
237	poricoic acid AM	$C_{32}H_{48}O_5$	511.34289	Poriacocos
238	acetyleburicoic acid	$C_{33}H_{52}O_4$	511.37928	Poriacocos
239	Mazatlanone	$C_{29}H_{22}O_9$	513.1186	Polygala
240	5α,8α-Peroxydehydrotumulosic acid	$C_{31}H_{46}O_6$	513.3219	Poriacocos
241	poricoic acid F	$C_{31}H_{46}O_6$	513.32216	Poriacocos
242	poricoic acid D	$C_{31}H_{46}O_6$	513.322162	Poriacocos
243	poricoic acid I	$C_{31}H_{46}O_6$	513.322162	Poriacocos
244	Poricoic acid GE	$C_{32}H_{50}O_5$	513.3565	Poriacocos
245	poricoic acid HM	$C_{32}H_{50}O_5$	513.3585	Poriacocos
246	3-O-acetyl-16α-hydroxytrametenolic acid	$C_{32}H_{50}O_5$	513.35854	Poriacocos
247	3−Acetyloxy−16α−hydroxytrametenolic acid	$C_{32}H_{50}O_5$	513.359	Poriacocos
248	5,8α−Dioxy−3β,16α−dihydroxyl−lanost−7(11), 24−dien−21−oic acid	$C_{31}H_{48}O_6$	515.3374	Poriacocos
249	25-hydroxyporicoic acid H	$C_{31}H_{48}O_6$	515.3378	Poriacocos
250	Arillanin B	$C_{22}H_{30}O_{14}$	517.1558	Polygala
251	Arillatose B	$C_{22}H_{30}O_{14}$	517.1558	Polygala
252	Sibiricose A5	$C_{22}H_{30}O_{14}$	517.1558	Polygala
253	Tenuifolioside A	$C_{22}H_{30}O_{14}$	517.1558	Polygala
254	Presenegenin	$C_{30}H_{46}O_7$	517.3166	Polygala
255	Ganoderic acid C2	$C_{30}H_{46}O_7$	517.31671	Poriacocos
256	poricoic acid M	$C_{30}H_{46}O_7$	517.31707	Poriacocos
257	Wubangziside A	$C_{24}H_{26}O_{13}$	521.1295	Polygala
258	Tricornoside C	$C_{24}H_{26}O_{13}$	521.1295	Polygala
259	Tricornoside F	$C_{24}H_{26}O_{13}$	521.1295	Polygala
260	16α-Acetyloxy-24-methylene-3-oxolanosta-7,9(11)-dien-21-oic acid	$C_{33}H_{48}O_5$	523.3425	Poriacocos
261	3−Oxo−16α−acetyloxylanosta−7,9(11), 24−trien−21−oic acid	$C_{33}H_{48}O_5$	523.344	Poriacocos
262	16α-Acetyloxyeburiconic acid	$C_{33}H_{50}O_5$	525.3565	Poriacocos
263	3-epi-dehydropachymic acid	$C_{33}H_{50}O_5$	525.3585	Poriacocos
264	poricoic acid AE	$C_{33}H_{50}O_5$	525.35854	Poriacocos
265	1-O-methylmazatlanone	$C_{30}H_{24}O_9$	527.1342	Polygala
266	poricoic acid DM	$C_{32}H_{48}O_6$	527.3378	Poriacocos
267	25-methoxyporicoic acid A	$C_{32}H_{48}O_6$	527.337812	Poriacocos
268	3-epi-Pachymic acid	$C_{33}H_{52}O_5$	527.3733	Poriacocos
269	Poricoic acid HE	$C_{33}H_{52}O_5$	527.3739	Poriacocos
270	pachymic acid	$C_{33}H_{52}O_5$	527.76	Poriacocos
271	poricoic acid J	$C_{31}H_{46}O_7$	529.31707	Poriacocos
272	poricoic acid L	$C_{31}H_{46}O_7$	529.31707	Poriacocos
273	3-O-Acetyl-16α,26-dihydroxytrametenolic acid	$C_{32}H_{50}O_6$	529.35309	Poriacocos
274	3β-Acetoxy-16α,26-dihydroxylanosta-8,24-di	$C_{32}H_{50}O_6$	529.35321	Poriacocos

	en-21-oic acid			
275	Tenuifolioside B	$C_{23}H_{32}O_{14}$	531.1714	Polygala
276	Tenuifolioside C	$C_{23}H_{32}O_{14}$	531.1714	Polygala
277	poricoic acid K	$C_{31}H_{48}O_{7}$	531.33272	Poriacocos
278	Polycaudoside A	$C_{25}H_{28}O_{13}$	535.1452	Polygala
279	Tricornoside D	$C_{25}H_{28}O_{13}$	535.1452	Polygala
280	Wattersiixanthone A	$C_{25}H_{28}O_{13}$	535.1452	Polygala
281	Sibiricaxanthone A	$C_{24}H_{26}O_{14}$	537.1245	Polygala
282	Sibiricaxanthone B	$C_{24}H_{26}O_{14}$	537.1245	Polygala
283	2'-benzoylmangiferin	$C_{26}H_{22}O_{13}$	541.0982	Polygala
284	6α-Hydroxydehydropachymic acid	$C_{33}H_{50}O_{6}$	541.35346	Poriacocos
285	29α-hydroxydehydrotumulosic acid	$C_{33}H_{50}O_{6}$	541.353462	Poriacocos
286	29-Hydroxydehydropachymic acid	$C_{33}H_{50}O_{6}$	541.3537	Poriacocos
287	pachymic acid methyl ester	$C_{34}H_{54}O_{5}$	541.3898	Poriacocos
288	26-hydroxyporicoic acid DM	$C_{32}H_{48}O_{7}$	543.33272	Poriacocos
289	6α−Hydroxydehydropachymic acid	$C_{32}H_{48}O_{7}$	543.3332	Poriacocos
290	25-hydroxypachymic acid	$C_{33}H_{52}O_{6}$	543.36911	Poriacocos
291	Arillanin C	$C_{23}H_{32}O_{15}$	547.1663	Polygala
292	Sibiricose A1	$C_{23}H_{32}O_{15}$	547.1663	Polygala
293	Sibiricose A6	$C_{23}H_{32}O_{15}$	547.1663	Polygala
294	1'-Sinapoylsucrose	$C_{23}H_{32}O_{15}$	547.1663	Polygala
295	Telephiose F	$C_{26}H_{30}O_{13}$	549.1608	Polygala
296	Telephioxanthone A	$C_{28}H_{24}O_{12}$	551.119	Polygala
297	Telephioxanthone B	$C_{28}H_{24}O_{12}$	551.119	Polygala
298	Polygalaxanthone IX	$C_{25}H_{28}O_{14}$	551.1401	Polygala
299	Arillanin E	$C_{25}H_{28}O_{14}$	551.1401	Polygala
300	7-Methoxy-1-methylmazatlanone	$C_{31}H_{26}O_{10}$	557.1448	Polygala
301	25,26-Dihydroxydehydropachymic acid	$C_{33}H_{50}O_{7}$	557.3481	Poriacocos
302	l'-Acetyl-3'-feruloylsucrose	$C_{24}H_{32}O_{15}$	559.1663	Polygala
303	25-Methoxy-29-hydroxyporicoic acid HM	$C_{33}H_{52}O_{7}$	559.36377	Poriacocos
304	Glomeratose A	$C_{24}H_{34}O_{15}$	561.182	Polygala
305	Sibiricose A2	$C_{24}H_{34}O_{15}$	561.182	Polygala
306	Arillanin D	$C_{26}H_{30}O_{14}$	565.1558	Polygala
307	Polygalaxanthone III	$C_{25}H_{28}O_{15}$	567.135	Polygala
308	Polygalaxanthone VIII	$C_{25}H_{28}O_{15}$	567.135	Polygala
309	Polygalaxanthone XI	$C_{25}H_{28}O_{15}$	567.135	Polygala
310	Wubangziside C	$C_{25}H_{28}O_{15}$	567.135	Polygala
311	3β,15α-Bis(acetyloxy)-24-methylenelanost-8-en-21-oic acid	$C_{35}H_{54}O_{6}$	569.3831	Poriacocos
312	16−O−acetyl−pachymic acid	$C_{35}H_{54}O_{6}$	569.3857	Poriacocos
313	5,8α−Epidioxy −3β−[(2−hydroxyacetyl) oxy] −16−hydroxylanosta−6,9(11),24−trien−21−oic acid	$C_{33}H_{48}O_{8}$	571.3281	Poriacocos
314	3β,16α-Bis(acetyloxy)-29-hydroxylanosta-8,2	$C_{34}H_{52}O_{7}$	571.3619	Poriacocos

	4-dien-21-oic acid			
315	β-daucosterol	$C_{35}H_{60}O_6$	575.43171	Poriacocos
316	Sibiricaxanthone E	$C_{26}H_{28}O_{15}$	579.135	Polygala
317	Tricornoside B	$C_{27}H_{32}O_{14}$	579.1714	Polygala
318	Polyhongkongenoside A	$C_{25}H_{26}O_{16}$	581.1143	Polygala
319	Polygalaxanthone V	$C_{26}H_{30}O_{15}$	581.1507	Polygala
320	3−epi−(3′−o−methyl malonyloxy) −dehydrotumulosic acid	$C_{35}H_{52}O_7$	583.3603	Poriacocos
321	O−acetylpachymic acid methyl ester	$C_{36}H_{56}O_6$	583.3941	Poriacocos
322	3β− Benzoyldehydrotumulosic acid	$C_{38}H_{52}O_5$	587.374	Poriacocos
323	Syringaresinol-4'-O-β-D-monoglucoside	$C_{29}H_{38}O_{13}$	593.2234	Polygala
324	Polygalaxanthone IV	$C_{27}H_{32}O_{15}$	595.1663	Polygala
325	Sibiricaxanthone D	$C_{27}H_{32}O_{15}$	595.1663	Polygala
326	Tricornose A	$C_{26}H_{36}O_{16}$	603.1925	Polygala
327	3β-p-Hydroxybenzoyldehydrotumulosic acid	$C_{38}H_{52}O_6$	603.3671	Poriacocos
328	Polygalaxanthone VII	$C_{27}H_{32}O_{16}$	611.1612	Polygala
329	3−epi−(3′−hydroxy−3′−methylglutaryloxyl) −16α−hydroxydehydrotrametenolic acid	$C_{36}H_{54}O_8$	613.3751	Poriacocos
330	3-epi-(3'-Hydroxy-3'-methylglutaryloxyl)-16 α-hydroxyeburicoic acid	$C_{36}H_{56}O_8$	615.3913	Poriacocos
331	Reiniose B	$C_{29}H_{34}O_{15}$	621.182	Polygala
332	Reiniose C	$C_{29}H_{34}O_{15}$	621.182	Polygala
333	Ginsenoside Rh_2	$C_{36}H_{62}O_8$	622.4445	Ginseng
334	3−epi−(3′−hydroxyl−3′−methylglutaryloxyl) −dehydrotumulosic acid	$C_{37}H_{56}O_8$	627.6017	Poriacocos
335	3−epi−(3′−hydroxyl−3′−methylglutaryloxyl) −tumulosic acid	$C_{37}H_{56}O_8$	629.4066	Poriacocos
336	α-L-Arabinopyranosyl-3β,23,27,29-tetrahydr oxyolean-12-en-28-oate	$C_{35}H_{56}O_{10}$	635.3795	Polygala
337	Sibiricaxanthone F	$C_{29}H_{34}O_{16}$	637.1769	Polygala
338	Polygalaxanthone X	$C_{29}H_{36}O_{16}$	639.1925	Polygala
339	Polygalasaponin XXV	$C_{36}H_{56}O_{10}$	647.3795	Polygala
340	Fallaxsaponin A	$C_{35}H_{54}O_{11}$	649.3588	Polygala
341	Bayogenin-3-O-β-D-glucopyranoside	$C_{36}H_{58}O_{10}$	649.3952	Polygala
342	3'-Sinapoyl-6-benzoylsucrose	$C_{30}H_{36}O_{16}$	651.1925	Polygala
343	3'-(3,4,5-Trimethoxycinnamoyl)-6-benzoylsu crose	$C_{31}H_{38}O_{16}$	665.2082	Polygala
344	3'-(3,4,5-Trimethoxycinnamoyl)-4-benzoylsu crose	$C_{31}H_{38}O_{16}$	665.2082	Polygala
345	29-[O-β-D-Glucopyranosyloxy]-3β,23,27-trih ydroxyolean-12-en-28-oic acid	$C_{36}H_{58}O_{11}$	665.3901	Polygala
346	Ginsenoside Rk_3/ Ginsenoside Rh_4	$C_{36}H_{60}O_8$	665.4259	Ginseng
347	Ginsenoside Rk_4	$C_{36}H_{60}O_8$	665.4277	Ginseng

348	Tenuifoliside B	$C_{30}H_{36}O_{17}$	667.1874	Polygala
349	Compound K	$C_{36}H_{62}O_{8}$	667.4417	Ginseng
350	Arillatose C	$C_{28}H_{40}O_{19}$	679.2086	Polygala
351	Arillatose E	$C_{28}H_{40}O_{19}$	679.2086	Polygala
352	Tenuifolin	$C_{36}H_{56}O_{12}$	679.3694	Polygala
353	3'-(3,4,5-Trimethoxycinnamoyl)-4-p-hydroxy benzoylsucrose	$C_{31}H_{38}O_{17}$	681.2031	Polygala
354	1'-O-(3,4,5-trimethoxycinnamoyl)-6-p-hydro xybenzoylsucrose	$C_{31}H_{38}O_{17}$	681.2031	Polygala
355	Tenuifoliside A	$C_{31}H_{38}O_{17}$	681.2031	Polygala
356	20(R)-Ginsenoside Rh_1	$C_{36}H_{62}O_{9}$	683.4365	Ginseng
357	Ginsenoside F_1	$C_{36}H_{62}O_{9}$	683.4376	Ginseng
358	Ginsenoside Rh_1	$C_{36}H_{62}O_{9}$	683.4376	Ginseng
359	Glomeratose B	$C_{32}H_{38}O_{17}$	693.2031	Polygala
360	3'-(3,4,5-Trimethoxycinnamoyl)-6-anisoylsuc rose	$C_{32}H_{40}O_{17}$	695.2187	Polygala
361	24(R)-Pseudoginsenoside Rt_5	$C_{36}H_{62}O_{10}$	699.4331	Ginseng
362	Glomeratose C	$C_{33}H_{40}O_{17}$	707.2187	Polygala
363	Tricornose B	$C_{33}H_{40}O_{17}$	707.2187	Polygala
364	Arillatose D	$C_{29}H_{42}O_{20}$	709.2191	Polygala
365	Arillatose F	$C_{29}H_{42}O_{20}$	709.2191	Polygala
366	Telephiose C	$C_{32}H_{40}O_{18}$	711.2137	Polygala
367	3'-Feruloy-6-sinapoylsucrose	$C_{33}H_{40}O_{18}$	723.2137	Polygala
368	Arillanin A	$C_{33}H_{40}O_{18}$	723.2137	Polygala
369	1'-Sinapoyl-3'-feruloylsucrose	$C_{33}H_{40}O_{18}$	723.2137	Polygala
370	Reiniose A	$C_{34}H_{42}O_{18}$	737.2293	Polygala
371	Glomeratose E	$C_{34}H_{40}O_{19}$	751.2086	Polygala
372	Arillatose A	$C_{34}H_{40}O_{19}$	751.2086	Polygala
373	3'-*O*-Sinapoyl-6-4-methyl-3,5-dimethoxycinn amoyl)	$C_{35}H_{44}O_{18}$	751.245	Polygala
374	Sibiricose A4	$C_{34}H_{42}O_{19}$	753.2242	Polygala
375	Telephiose E	$C_{34}H_{42}O_{19}$	753.2242	Polygala
376	3',6-Disinapoylsucrose	$C_{34}H_{42}O_{19}$	753.2242	Polygala
377	3,6'-Disinapoylsucrose	$C_{34}H_{42}O_{19}$	753.2242	Polygala
378	1',3'-Disinapoylsucrose	$C_{34}H_{42}O_{19}$	753.2242	Polygala
379	Pseudo-ginsenoside Rp_1	$C_{41}H_{64}O_{13}$	763.42688	Ginseng
380	Gmsenoside F_4/Rg_4	$C_{42}H_{70}O_{12}$	765.476	Ginseng
381	Telephiose G	$C_{34}H_{40}O_{20}$	767.2035	Polygala
382	Tenuifoliside C	$C_{35}H_{44}O_{19}$	767.2399	Polygala
383	Notoginsenoside R_2	$C_{41}H_{70}O_{13}$	769.4746	Ginseng
384	Ginsenoside F_3	$C_{41}H_{70}O_{13}$	769.4749	Ginseng
385	3',6'-Diferuloyl-4,6-diacetylsucrose	$C_{36}H_{42}O_{19}$	777.2242	Polygala
386	3',6'-Diferuloyl-3,6-diacetylsucrose	$C_{36}H_{44}O_{19}$	777.2242	Polygala
387	Polygalasaponin XXVII	$C_{41}H_{64}O_{14}$	779.4218	Polygala

388	Glomeratose D	$C_{36}H_{46}O_{19}$	781.2555	Polygala
389	Ginsenoside F_2	$C_{42}H_{72}O_{13}$	783.49	Ginseng
390	Pseudogisnenoside RT_2	$C_{41}H_{70}O_{14}$	785.4693	Ginseng
391	Zingibroside R_1	$C_{42}H_{66}O_{14}$	793.4379	Ginseng
392	Chikusetsusaponin Iva	$C_{42}H_{66}O_{14}$	793.4399	Ginseng
393	3-Acetyl-3',6-*O*-disinapoylsucrose	$C_{36}H_{44}O_{20}$	795.2348	Polygala
394	4-Acetyl-3',6-*O*-disinapoylsucrose	$C_{36}H_{44}O_{20}$	795.2348	Polygala
395	Telephiose A	$C_{36}H_{44}O_{20}$	795.2348	Polygala
396	Telephiose B	$C_{36}H_{44}O_{20}$	795.2348	Polygala
397	Hederagenin 3-*O*-β-D-glucopyranosyl (1→2) β-D-glucopyranoside	$C_{42}H_{68}O_{14}$	795.4531	Polygala
398	Polygalasaponin XX	$C_{42}H_{68}O_{14}$	795.4531	Polygala
399	Notoginsenoside T_5	$C_{41}H_{68}O_{12}$	797.4683	Ginseng
400	Sibiricaxanthone G	$C_{35}H_{44}O_{21}$	799.2297	Polygala
401	3'-Feruloyl-6'-sinapoyl-4,6-diacetylsucrose	$C_{37}H_{44}O_{20}$	807.2348	Polygala
402	Ginsenoside Rs_4/Rs_5	$C_{44}H_{72}O_{13}$	807.4903	Ginseng
403	Polygalasaponin XII	$C_{42}H_{66}O_{15}$	809.4324	Polygala
404	Polygalasaponin XIII	$C_{42}H_{66}O_{15}$	809.4324	Polygala
405	Polygalasaponin XXVI	$C_{42}H_{66}O_{15}$	809.4324	Polygala
406	Polygalasaponin I	$C_{42}H_{68}O_{15}$	811.448	Polygala
407	Lobatoside B	$C_{42}H_{68}O_{15}$	811.448	Polygala
408	Polygalasaponin C	$C_{42}H_{68}O_{15}$	811.448	Polygala
409	Polygalasaponin D	$C_{42}H_{68}O_{15}$	811.448	Polygala
410	Ginsenoside Rg_6	$C_{42}H_{70}O_{12}$	811.4849	Ginseng
411	Ginsenoside Rg_4	$C_{42}H_{70}O_{12}$	811.4852	Ginseng
412	Ginsenoside Rg_5	$C_{42}H_{70}O_{12}$	811.4852	Ginseng
413	Ginsenoside Rk_1	$C_{42}H_{70}O_{12}$	811.4852	Ginseng
414	Ginsenoside F_4	$C_{42}H_{70}O_{12}$	811.4865	Ginseng
415	Ginsenoside F_3	$C_{41}H_{70}O_{13}$	815.4788	Ginseng
416	Ginsenoside F_5	$C_{41}H_{70}O_{13}$	815.4788	Ginseng
417	Pseudoginsenoside RT_3	$C_{41}H_{70}O_{13}$	815.4788	Ginseng
418	Compound II	$C_{41}H_{70}O_{13}$	815.479	Ginseng
419	Floralginsenoside B	$C_{42}H_{72}O_{15}$	815.4798	Ginseng
420	20(R)-Notoginsenoside R_2	$C_{41}H_{70}O_{13}$	815.481	Ginseng
421	Sanchinoside A3	$C_{41}H_{70}O_{13}$	815.4819	Ginseng
422	Majonoside R_1/isomer	$C_{42}H_{72}O_{15}$	816.4818	Ginseng
423	Dalmaisiose A	$C_{38}H_{46}O_{20}$	821.2504	Polygala
424	Ginsenoside Rs_3	$C_{44}H_{74}O_{14}$	825.5012	Ginseng
425	Ginsenoside Rg_{10} / Ginsenoside Rg_9	$C_{42}H_{70}O_{13}$	827.4788	Ginseng
426	20(R)-Ginsenoside Rg_2	$C_{42}H_{72}O_{13}$	829.4944	Ginseng
427	Gmsenoside F_2	$C_{42}H_{72}O_{13}$	829.4962	Ginseng
428	20(R)-Ginsenoside Rg_3	$C_{42}H_{72}O_{13}$	829.4965	Ginseng
429	Ginsenoside Rg_2	$C_{42}H_{72}O_{13}$	829.4966	Ginseng
430	Telephiose D	$C_{38}H_{46}O_{21}$	837.2454	Polygala

431	Reinioside A	$C_{42}H_{66}O_{17}$	841.4222	Polygala
432	Notoginsenoside Rt	$C_{44}H_{74}O_{15}$	841.49591	Ginseng
433	Ginsenoside Rf	$C_{42}H_{72}O_{14}$	845.4815	Ginseng
434	Ginsenoside Rg_1	$C_{42}H_{72}O_{14}$	845.4893	Ginseng
435	Pseudoginsenoside F_{11}	$C_{42}H_{72}O_{14}$	845.4904	Ginseng
436	Butenyl group- Rf	$C_{46}H_{76}O_{15}$	868.5179	Ginseng
437	Butenyl group- Rg_1	$C_{46}H_{76}O_{15}$	868.5179	Ginseng
438	Reiniose D	$C_{42}H_{48}O_{20}$	871.2661	Polygala
439	Ginsenoside Rs_3	$C_{44}H_{74}O_{14}$	871.505	Ginseng
440	Reiniose E	$C_{39}H_{50}O_{23}$	885.2665	Polygala
441	Notoginsenoside Rt	$C_{44}H_{74}O_{15}$	887.4988	Ginseng
442	4'-O-acetyl-pseudoginsenoside F_{11}	$C_{44}H_{74}O_{15}$	887.4999	Ginseng
443	6'-O-acetyl-ginsenoside Rg_1	$C_{44}H_{74}O_{15}$	887.4999	Ginseng
444	Tenuifoliside E	$C_{41}H_{48}O_{22}$	891.2559	Polygala
445	Reiniose F	$C_{40}H_{52}O_{24}$	915.2771	Polygala
446	Tricornose D	$C_{40}H_{52}O_{24}$	915.2771	Polygala
447	Pseudoginsenoside Rt_1	$C_{47}H_{74}O_{18}$	925.4783	Ginseng
448	Chikusetsusaponin Iv	$C_{47}H_{74}O_{18}$	925.4795	Ginseng
449	Polygalasaponin A	$C_{47}H_{76}O_{18}$	927.4954	Polygala
450	Tricornose C	$C_{41}H_{54}O_{24}$	929.2927	Polygala
451	Ginsenoside Re_4	$C_{47}H_{80}O_{18}$	931.5267	Ginseng
452	Notoginsenoside R_1	$C_{47}H_{80}O_{18}$	931.52692	Ginseng
453	Polygalasaponin E	$C_{47}H_{74}O_{19}$	941.4746	Polygala
454	Ginsenoside Rd	$C_{48}H_{82}O_{18}$	945.5399	Ginseng
455	Ginsenoside Re	$C_{48}H_{82}O_{18}$	945.5448	Ginseng
456	Gypenoside XVII	$C_{48}H_{82}O_{18}$	946.5501	Ginseng
457	Ginsenoside Ro	$C_{48}H_{76}O_{19}$	955.4917	Ginseng
458	Polygalasaponin II	$C_{48}H_{78}O_{19}$	957.5059	Polygala
459	Ginsenoside Re_2	$C_{48}H_{82}O_{19}$	961.5347	Ginseng
460	Ginsenoside Re_3	$C_{48}H_{82}O_{19}$	961.5347	Ginseng
461	Ginsenoside Re_1	$C_{48}H_{82}O_{19}$	961.5358	Ginseng
462	20-glc-Rf	$C_{48}H_{82}O_{19}$	962.5385	Ginseng
463	Compound Mc_1	$C_{47}H_{80}O_{17}$	962.545	Ginseng
464	Gypenoside IX	$C_{47}H_{80}O_{17}$	962.545	Ginseng
465	Notoginsenoside N_1/isomer	$C_{48}H_{82}O_{19}$	962.545	Ginseng
466	Polygalasaponin XLVIII	$C_{48}H_{76}O_{20}$	971.4852	Polygala
467	Polygalasaponin XIV	$C_{48}H_{76}O_{20}$	971.4852	Polygala
468	Polygalasaponin XLVII	$C_{48}H_{76}O_{20}$	971.4852	Polygala
469	Polygalasaponin VI	$C_{48}H_{78}O_{20}$	973.5008	Polygala
470	Polygalasaponin B	$C_{48}H_{78}O_{20}$	973.5008	Polygala
471	Notoginsenoside Fp_1	$C_{47}H_{80}O_{18}$	977.53284	Ginseng
472	Pseudoginsenoside Rc_1	$C_{50}H_{84}O_{19}$	988.5607	Ginseng
473	Gypenoside XVII	$C_{48}H_{82}O_{18}$	991.5472	Ginseng
474	Notoginsenoside K	$C_{48}H_{82}O_{18}$	991.5504	Ginseng

475	20-O-Glucosylginsenoside Rf	$C_{48}H_{82}O_{19}$	1007.5408	Ginseng
476	Notoginsenoside R_3	$C_{48}H_{82}O_{19}$	1007.5421	Ginseng
477	Notoginsenoside N	$C_{48}H_{82}O_{19}$	1007.54279	Ginseng
478	VInaginsenoSide R_4	$C_{48}H_{82}O_{19}$	1007.5456	Ginseng
479	Bu-Gypenoside XVII	$C_{52}H_{86}O_{19}$	1014.5757	Ginseng
480	Butenyl group -Rd	$C_{52}H_{86}O_{19}$	1014.5757	Ginseng
481	Butenyl group - Re	$C_{52}H_{86}O_{19}$	1014.5758	Ginseng
482	Malonyl-floralginsenoside Re_1	$C_{51}H_{84}O_{21}$	1031.54321	Ginseng
483	Malonyl-ginsenoside XDII	$C_{51}H_{84}O_{21}$	1031.54346	Ginseng
484	Malonyi-Ginsenoside Rd	$C_{51}H_{84}O_{21}$	1031.5435	Ginseng
485	Malonyl-ginsenoside Re	$C_{51}H_{84}O_{21}$	1031.5437	Ginseng
486	Malonyl-Floralginsenoside Rd_5	$C_{51}H_{84}O_{21}$	1031.5453	Ginseng
487	Fallaxose A	$C_{48}H_{58}O_{25}$	1033.3189	Polygala
488	Polygalasaponin G	$C_{52}H_{84}O_{22}$	1059.5376	Polygala
489	Polygalasaponin J	$C_{52}H_{82}O_{23}$	1073.5169	Polygala
490	Fallaxose B	$C_{50}H_{60}O_{26}$	1075.3295	Polygala
491	Watterose D	$C_{49}H_{58}O_{27}$	1077.3087	Polygala
492	Watterose I	$C_{49}H_{58}O_{27}$	1077.3087	Polygala
493	Tricornose G	$C_{46}H_{62}O_{29}$	1077.3299	Polygala
494	Ginsenoside Rc	$C_{53}H_{90}O_{22}$	1077.5833	Ginseng
495	Ginsenoside Rb_3	$C_{53}H_{90}O_{22}$	1077.5838	Ginseng
496	Ginsenoside Rb_2	$C_{53}H_{90}O_{22}$	1077.5839	Ginseng
497	Polygalasaponin III	$C_{53}H_{86}O_{23}$	1089.5482	Polygala
498	Polygalasaponin F	$C_{53}H_{86}O_{23}$	1089.5482	Polygala
499	Notoginsenoside O/P	$C_{52}H_{88}O_{21}$	1093.5813	Ginseng
500	Polygalasaponin XXIII	$C_{53}H_{82}O_{24}$	1101.5118	Polygala
501	Crotalarioside A	$C_{53}H_{82}O_{24}$	1101.5118	Polygala
502	Amarelloside	$C_{51}H_{60}O_{27}$	1103.3244	Polygala
503	Fallaxose C	$C_{51}H_{60}O_{27}$	1103.3244	Polygala
504	Watterose H	$C_{51}H_{60}O_{27}$	1103.3244	Polygala
505	Polygalasaponin XXVIII	$C_{53}H_{84}O_{24}$	1103.5275	Polygala
506	Polygalasaponin XXI	$C_{53}H_{84}O_{24}$	1103.5275	Polygala
507	Tricornose F	$C_{51}H_{62}O_{27}$	1105.34	Polygala
508	Ginsenoside Rb_1	$C_{54}H_{92}O_{23}$	1107.5957	Ginseng
509	Polygalasaponin XVII	$C_{54}H_{86}O_{24}$	1117.5431	Polygala
510	Ginsenoside Roa	$C_{54}O_{86}O_{24}$	1117.54382	Ginseng
511	Watterose C	$C_{51}H_{60}O_{28}$	1119.3193	Polygala
512	Polygalasaponin VII	$C_{54}H_{88}O_{24}$	1119.5588	Polygala
513	Ginsenoside Rs_2	$C_{55}H_{92}O_{23}$	1119.5955	Ginseng
514	Ginsenoside Rs_1	$C_{55}H_{92}O_{23}$	1119.5973	Ginseng
515	Ginsenoside Rb_2	$C_{53}H_{90}O_{22}$	1123.5895	Ginseng
516	Ginsenoside Rb_3	$C_{53}H_{90}O_{22}$	1123.5895	Ginseng
517	Notoginsenoside A/ isomer	$C_{54}H_{92}O_{24}$	1124.5979	Ginseng
518	Fallaxose D	$C_{52}H_{62}O_{28}$	1133.335	Polygala

519	Watterose A	$C_{52}H_{62}O_{28}$	1133.335	Polygala
520	Onjisaponin I	$C_{54}H_{86}O_{25}$	1133.538	Polygala
521	Polygalasaponin XV	$C_{54}H_{86}O_{25}$	1133.538	Polygala
522	Watterose J	$C_{51}H_{60}O_{29}$	1135.3142	Polygala
523	Tricornose E	$C_{52}H_{64}O_{28}$	1135.3506	Polygala
524	Polygalasaponin XI	$C_{54}H_{88}O_{25}$	1135.5537	Polygala
525	Notoginsenoside C	$C_{54}H_{92}O_{25}$	1139.58301	Ginseng
526	Reiniose G	$C_{53}H_{62}O_{28}$	1145.335	Polygala
527	Polygalasaponin XXXIII	$C_{55}H_{86}O_{25}$	1145.538	Polygala
528	ginsenoside Ra_7	$C_{57}H_{94}O_{23}$	1146.618	Ginseng
529	ginsenoside Ra_8/Ra_9	$C_{57}H_{94}O_{23}$	1146.618	Ginseng
530	Watterose F	$C_{52}H_{62}O_{29}$	1149.3299	Polygala
531	Quinquenoside R_1	$C_{56}H_{94}O_{24}$	1150.6135	Ginseng
532	5,6-Didehydroginsenoside Rb_1	$C_{54}H_{90}O_{23}$	1151.5861	Ginseng
533	Ginsenoside Rb_1	$C_{54}H_{92}O_{23}$	1153.6031	Ginseng
534	Watterose B	$C_{53}H_{62}O_{29}$	1161.3299	Polygala
535	Senegose G	$C_{53}H_{64}O_{29}$	1163.3455	Polygala
536	Senegose H	$C_{53}H_{64}O_{29}$	1163.3455	Polygala
537	Malonyl-ginsenoside Rb_2	$C_{56}H_{92}O_{25}$	1163.58484	Ginseng
538	Malonyl-ginsenoside Rc	$C_{56}H_{92}O_{25}$	1163.58484	Ginseng
539	Malonyl-Rb3	$C_{56}H_{92}O_{25}$	1164.5928	Ginseng
540	Glomeratose F	$C_{54}H_{64}O_{29}$	1175.3455	Polygala
541	Reiniose H	$C_{54}H_{64}O_{29}$	1175.3455	Polygala
542	ginsenoside Ra_6	$C_{58}H_{96}O_{24}$	1176.6075	Ginseng
543	Watterose G	$C_{53}H_{62}O_{30}$	1177.3248	Polygala
544	Arilloside A	$C_{57}H_{88}O_{26}$	1187.5486	Polygala
545	Watterose E	$C_{54}H_{64}O_{30}$	1191.3404	Polygala
546	Malonyi-Ginsenoside Rb_1	$C_{57}H_{94}O_{26}$	1193.5999	Ginseng
547	Quinquenoside R_1	$C_{56}H_{94}O_{24}$	1195.6106	Ginseng
548	Senegose F	$C_{55}H_{66}O_{30}$	1205.3561	Polygala
549	Senegose I	$C_{55}H_{66}O_{30}$	1205.3561	Polygala
550	Ginsenoside Ra_2	$C_{58}H_{98}O_{26}$	1209.6262	Ginseng
551	Ginsenoside Ra_1	$C_{58}H_{98}O_{26}$	1209.6272	Ginseng
552	Notoginsenoside Fc	$C_{58}H_{98}O_{26}$	1210.6223	Ginseng
553	Polygalasaponin IV	$C_{58}H_{94}O_{27}$	1221.5904	Polygala
554	Polygalasaponin V	$C_{58}H_{94}O_{27}$	1221.5904	Polygala
555	Tricornose L	$C_{55}H_{68}O_{31}$	1223.3667	Polygala
556	3-O-β-D-Glucopyranosyl 2-oxo-olean-12-en-23,28-dioic acid 28-O-{β-D-xylopyranosyl(1→4)-[β-D-apiofuranosyl(1→3)]-α-L-rhamnopyranosyl(1→2)-β-D-glucopyranosyl} ester	$C_{58}H_{90}O_{28}$	1233.5541	Polygala
557	Arilloside B	$C_{58}H_{92}O_{28}$	1235.5697	Polygala
558	Arillatanoside A	$C_{58}H_{92}O_{28}$	1235.5697	Polygala

559	Polygalasaponin XXII	$C_{58}H_{92}O_{28}$	1235.5697	Polygala
560	3-O-β-D-Glucopyranosyl medicagenic acid 28-O-{β-D-xylopyranosyl(1→4)-[β-D-apiofuranosyl (1→3)]-α-L-rhamnopyranosyl(1→2)-β-D-glucopyranosyl} ester	$C_{58}H_{92}O_{28}$	1235.5697	Polygala
561	Dalmaisiose J	$C_{59}H_{66}O_{29}$	1237.3612	Polygala
562	Notoginsenoside Fa/R4	$C_{59}H_{100}O_{27}$	1239.63757	Ginseng
563	Ginsenoside Ra3	$C_{59}H_{100}O_{27}$	1239.6377	Ginseng
564	GinsenosideR4	$C_{59}H_{100}O_{27}$	1239.6379	Ginseng
565	Dalmaisiose H	$C_{60}H_{66}O_{29}$	1249.3612	Polygala
566	Dalmaisiose D	$C_{57}H_{70}O_{31}$	1249.3823	Polygala
567	Polygalasaponin XVIII	$C_{59}H_{94}O_{28}$	1249.5854	Polygala
568	Micranthoside A	$C_{59}H_{94}O_{28}$	1249.5854	Polygala
569	Polygalasaponin XXIV	$C_{58}H_{92}O_{29}$	1251.5646	Polygala
570	Polygalasaponin VIII	$C_{59}H_{96}O_{28}$	1251.601	Polygala
571	Polygalasaponin IX	$C_{59}H_{96}O_{28}$	1251.601	Polygala
572	Polygalasaponin H	$C_{59}H_{96}O_{28}$	1251.601	Polygala
573	Tricornose K	$C_{56}H_{70}O_{32}$	1253.3772	Polygala
574	Ginsenoside Ra2	$C_{58}H_{98}O_{26}$	1255.6291	Ginseng
575	Crotalarioside B	$C_{59}H_{92}O_{29}$	1263.5646	Polygala
576	Tenuifoliose K	$C_{57}H_{70}O_{32}$	1265.3772	Polygala
577	Desacylsenegasaponin B	$C_{59}H_{94}O_{29}$	1265.5803	Polygala
578	3-O-(β-D-Glucopyranosyl) presenegenin 28-[O-β-D-galactopyranosyl-(1→4)-O-β-D-xylopyranosyl-(1→4)-O-α-L-rhamnopyranosyl-(1→2)-β-D-fucopyranosyl] ester	$C_{59}H_{94}O_{29}$	1265.5803	Polygala
579	Polygalasaponin XVI	$C_{59}H_{94}O_{29}$	1265.5803	Polygala
580	Dalmaisiose L	$C_{60}H_{68}O_{30}$	1267.3717	Polygala
581	Tricornose J	$C_{57}H_{72}O_{32}$	1267.3929	Polygala
582	Dalmaisiose E	$C_{58}H_{72}O_{32}$	1279.3929	Polygala
583	Dalmaisiose F	$C_{58}H_{72}O_{32}$	1279.3929	Polygala
584	Tricornose H	$C_{57}H_{72}O_{33}$	1283.3878	Polygala
585	Senegose D	$C_{57}H_{72}O_{33}$	1283.3878	Polygala
586	Notoginsenoside R4	$C_{59}H_{100}O_{27}$	1285.6453	Ginseng
587	Dalmaisiose B	$C_{59}H_{72}O_{32}$	1291.3929	Polygala
588	Micranthoside B	$C_{61}H_{96}O_{29}$	1291.5959	Polygala
589	Polygalasaponin XLII	$C_{64}H_{94}O_{27}$	1293.5904	Polygala
590	Senegose K	$C_{58}H_{72}O_{33}$	1295.3878	Polygala
591	Senegose M	$C_{58}H_{72}O_{33}$	1295.3878	Polygala
592	Tenuifoliose C	$C_{58}H_{72}O_{33}$	1295.3878	Polygala
593	Tenuifoliose E	$C_{58}H_{72}O_{33}$	1295.3878	Polygala
594	Tricornose I	$C_{58}H_{74}O_{33}$	1297.4034	Polygala
595	Tenuifoliose I	$C_{59}H_{72}O_{33}$	1307.3878	Polygala

596	Tenuifoliose J	$C_{59}H_{72}O_{33}$	1307.3878	Polygala
597	Polygalasaponin XXXIV	$C_{61}H_{96}O_{30}$	1307.5908	Polygala
598	Reinioside B	$C_{61}H_{96}O_{30}$	1307.5908	Polygala
599	Polygalasaponin XXXVII	$C_{61}H_{96}O_{30}$	1307.5908	Polygala
600	Dalmaisiose M	$C_{62}H_{70}O_{31}$	1309.3823	Polygala
601	Dalmaisiose G	$C_{59}H_{74}O_{33}$	1309.4034	Polygala
602	Arilloside C	$C_{62}H_{96}O_{30}$	1319.5908	Polygala
603	Fallaxose E	$C_{59}H_{74}O_{34}$	1325.3983	Polygala
604	Senegose B	$C_{59}H_{74}O_{34}$	1325.3983	Polygala
605	Senegose C	$C_{59}H_{74}O_{34}$	1325.3983	Polygala
606	Tenuifoliose P	$C_{59}H_{74}O_{34}$	1325.3983	Polygala
607	Reiniose J	$C_{60}H_{74}O_{34}$	1337.3983	Polygala
608	Senegose J	$C_{60}H_{74}O_{34}$	1337.3983	Polygala
609	Senegose L	$C_{60}H_{74}O_{34}$	1337.3983	Polygala
610	Senegose N	$C_{60}H_{74}O_{34}$	1337.3983	Polygala
611	Senegose O	$C_{60}H_{74}O_{34}$	1337.3983	Polygala
612	Tenuifoliose B	$C_{60}H_{74}O_{34}$	1337.3983	Polygala
613	Tenuifoliose D	$C_{60}H_{74}O_{34}$	1337.3983	Polygala
614	Notoginsenoside S	$C_{63}H_{106}O_{30}$	1341.6686	Ginseng
615	Tenuifoliose H	$C_{61}H_{74}O_{34}$	1349.3983	Polygala
616	Polygalasaponin XXXV	$C_{63}H_{98}O_{31}$	1349.6014	Polygala
617	Reinioside C	$C_{63}H_{98}O_{31}$	1349.6014	Polygala
618	Dalmaisiose C	$C_{61}H_{76}O_{34}$	1351.414	Polygala
619	28-O-[α-L-Rhamnopyranosyl(1→4)-α-L-rhamnopyranosyl(1→4)-[β-D-xylofuranosyl(1→2)-β-D-xylopyranosyl(1→3)]-β-D-xylopyranosyl]-3-O-β-D-glucopyranosyl-16α-hydroxy medicagenate	$C_{63}H_{100}O_{31}$	1351.6171	Polygala
620	Reiniose I	$C_{61}H_{76}O_{35}$	1367.4089	Polygala
621	Senegose A	$C_{61}H_{76}O_{35}$	1367.4089	Polygala
622	Senegose E	$C_{61}H_{76}O_{35}$	1367.4089	Polygala
623	28-O-[β-D-Glucofuranosyl(1→2)-β-D-xylopyranosyl(1→4)-α-L-rhamnopyranosyl(1→4)-[α-D-xylopyranosyl(1→3)]-β-D-xylopyranosyl]-3-O-β-D-glucopyranosyl-medicagenate	$C_{63}H_{100}O_{32}$	1367.612	Polygala
624	Notoginsenoside T	$C_{64}H_{108}O_{31}$	1371.6792	Ginseng
625	Tenuifoliose A	$C_{62}H_{76}O_{35}$	1379.4089	Polygala
626	Polygalasaponin XIX	$C_{64}H_{102}O_{32}$	1381.6276	Polygala
627	Polygalasaponin X	$C_{64}H_{104}O_{32}$	1383.6433	Polygala
628	3-O-(β-D-Glucopyranosyl) presenegenin 28-{O-α-L-arabinopyranosyl-(1→4)-O-β-D-xylopyranosyl-(1→4)-O-α-L-rhamnopyranosyl-(1→2)-4-O- [(E, Z)-4-methoxycinnamoyl]-β-D-fucopyranosyl	$C_{68}H_{100}O_{30}$	1395.6221	Polygala

	} ester			
629	Myrtifolioside D1/D2	$C_{68}H_{100}O_{30}$	1395.6221	Polygala
630	Myrtifolioside E1/E2	$C_{68}H_{100}O_{30}$	1395.6221	Polygala
631	Arilloside D	$C_{64}H_{102}O_{33}$	1397.6225	Polygala
632	Polygalasaponin XXIX	$C_{64}H_{102}O_{33}$	1397.6225	Polygala
633	Desacylsenegasaponin A	$C_{64}H_{102}O_{33}$	1397.6225	Polygala
634	Arillatanoside C	$C_{64}H_{102}O_{33}$	1397.6225	Polygala
635	3-β-O-D-Glucopyranosyl-(1→2)-β-D-glucopyranosyl presenegenin 28-O-β-D-xylopyranosyl (1→4)-[β-D-apiofuranosyl (1→3)]-α-L-rhamnopyranosyl (1→2)-β-D-fucopyranosyl ester	$C_{64}H_{102}O_{33}$	1397.6225	Polygala
636	Tenuifoliose O	$C_{63}H_{78}O_{36}$	1409.4195	Polygala
637	Tenuifoliose N	$C_{63}H_{78}O_{36}$	1409.4195	Polygala
638	Onjisaponin M	$C_{69}H_{102}O_{30}$	1409.6378	Polygala
639	Polygalasaponin L	$C_{64}H_{100}O_{34}$	1411.6018	Polygala
640	Desacylsenegin III	$C_{65}H_{104}O_{33}$	1411.6382	Polygala
641	Polygalasaponin XLIX	$C_{64}H_{102}O_{34}$	1413.6175	Polygala
642	Dalmaisiose I	$C_{67}H_{78}O_{34}$	1425.4296	Polygala
643	E-Senegasaponin B	$C_{69}H_{102}O_{31}$	1425.6327	Polygala
644	Z-Senegasaponin B	$C_{69}H_{102}O_{31}$	1425.6327	Polygala
645	3-O-(β-D-Glucopyranosyl) presenegenin 28-(O-β-D-glucopyranosyl-(1→3)-O-α-L-arabinopyranosyl-(1→4)-O-α-L-rhamnopyranosyl-(1→2)-{4-O-[(E, Z)-4-methoxycinnamoyl]}-β-D-fucopyranosyl) ester	$C_{69}H_{102}O_{31}$	1425.6327	Polygala
646	3-O-(β-D-Glucopyranosyl) presenegenin 28-{O-α-L-arabinopyranosyl-(1→4)-O-β-D-xylopyranosyl-(1→4)-O-α-L-rhamnopyranosyl-(1→2)-4-O-[(E)-3,4-dimethoxycinnamoyl]-β-D-fucopyranosyl} ester	$C_{69}H_{102}O_{31}$	1425.6327	Polygala
647	Myrtifolioside C1/ C2	$C_{69}H_{102}O_{31}$	1425.6327	Polygala
648	Desacylsenegasaponin C	$C_{65}H_{104}O_{34}$	1427.6331	Polygala
649	3-O-(β-D-Glucopyranosyl) presenegenin 28-{O-β-D-galactopyranosyl-(1→4)-O-[β-D-glucopyranosyl-(1→3)]-O-β-D-xylopyranosyl-(1→4)-O-α-L-rhamnopyranosyl-(1→2)- β-D-fucopyranosyl} ester	$C_{65}H_{104}O_{34}$	1427.6331	Polygala
650	3-O-β-D-Glucopyranosyl presenegenin-28-O-β-D-galactopyranosyl-(1→3)- β-D-xylopyranosyl-(1→4)- α-L-rhamnopyranosyl-(1→2)-[β-D-glucopyr	$C_{65}H_{104}O_{34}$	1427.6331	Polygala

	anosyl-(1→3)]- β-D-fucopyranoside			
651	Arilloside E	$C_{66}H_{104}O_{34}$	1439.6331	Polygala
652	Arillatanoside B	$C_{66}H_{104}O_{34}$	1439.6331	Polygala
653	Glomeratose G	$C_{67}H_{78}O_{35}$	1441.4246	Polygala
654	Dalmaisiose P	$C_{67}H_{80}O_{35}$	1443.4402	Polygala
655	Tenuifoliose M	$C_{65}H_{82}O_{37}$	1453.4457	Polygala
656	Tenuifoliose Q	$C_{65}H_{82}O_{37}$	1453.4457	Polygala
657	Polygalasaponin XXXIX	$C_{67}H_{106}O_{34}$	1453.6488	Polygala
658	Dalmaisiose K	$C_{68}H_{80}O_{35}$	1455.4402	Polygala
659	Dalmaisiose N	$C_{68}H_{80}O_{35}$	1455.4402	Polygala
660	E-Senegin II	$C_{70}H_{104}O_{32}$	1455.6433	Polygala
661	Z-Senegin II	$C_{70}H_{104}O_{32}$	1455.6433	Polygala
662	Onjisaponin G	$C_{70}H_{104}O_{32}$	1455.6433	Polygala
663	3-O-(β-D-Glucopyranosyl) presenegenin 28-(O-β-D-glucopyranosyl-(1→3)-O-α-L-arabinopyranosyl-(1→4)-O-α-L-rhamnopyranosyl-(1→2)-{4-O-[(E, Z)-3,4-dimethoxycinnamoyl]}-β-D-fucopyranosyl) ester	$C_{70}H_{104}O_{32}$	1455.6433	Polygala
664	Polygalasaponin XXXVIII	$C_{67}H_{106}O_{35}$	1469.6437	Polygala
665	Onjisaponin Z	$C_{71}H_{106}O_{32}$	1469.6589	Polygala
666	Arilloside F	$C_{68}H_{106}O_{35}$	1481.6437	Polygala
667	Reinioside D	$C_{68}H_{106}O_{35}$	1481.6437	Polygala
668	Tenuifoliose G	$C_{66}H_{84}O_{38}$	1483.4563	Polygala
669	Dalmaisiose O	$C_{69}H_{82}O_{36}$	1485.4508	Polygala
670	Onjisaponin E	$C_{71}H_{106}O_{33}$	1485.6538	Polygala
671	Tenuifoliose L	$C_{67}H_{84}O_{38}$	1495.4563	Polygala
672	Reinioside F	$C_{69}H_{108}O_{36}$	1511.6542	Polygala
673	3-O-β-D-Glucopyranosyl presenegenin 28-O-α-L-arabipyranosyl (1→3)-β-D-xylopyranosyl(1→4)-[β-D-apiofuranosyl(1→3)]-α-L-rhamnopyranosyl(1→2)-[α-L-rhamnopyranosyl(1→3)]-β-D-fucopyranosyl ester	$C_{69}H_{110}O_{36}$	1513.6699	Polygala
674	Polygalasaponin XXXVI	$C_{70}H_{108}O_{36}$	1523.6542	Polygala
675	Reinioside E	$C_{70}H_{108}O_{36}$	1523.6542	Polygala
676	Tenuifoliose F	$C_{68}H_{86}O_{39}$	1525.4668	Polygala
677	Arillatanoside D	$C_{69}H_{110}O_{37}$	1529.6648	Polygala
678	E-Onjisaponin H	$C_{74}H_{110}O_{34}$	1541.6801	Polygala
679	Z-Onjisaponin H	$C_{74}H_{110}O_{34}$	1541.6801	Polygala
680	Onjisaponin Pg	$C_{70}H_{110}O_{37}$	1545.675	Polygala
681	Polygalasaponin XLIII	$C_{74}H_{110}O_{35}$	1557.675	Polygala
682	E-Senegasaponin A	$C_{74}H_{110}O_{35}$	1557.675	Polygala
683	Z-Senegasaponin A	$C_{74}H_{110}O_{35}$	1557.675	Polygala

684	Myrtifolioside B1	$C_{74}H_{110}O_{35}$	1557.675	Polygala
685	Myrtifolioside B2	$C_{74}H_{110}O_{35}$	1557.675	Polygala
686	E-Senegin III	$C_{75}H_{112}O_{35}$	1571.6906	Polygala
687	Z-Senegin III	$C_{75}H_{112}O_{35}$	1571.6906	Polygala
688	Onjisaponin B	$C_{75}H_{112}O_{35}$	1571.6906	Polygala
689	Polygalasaponin XXX	$C_{75}H_{112}O_{36}$	1587.6855	Polygala
690	Polygalasaponin XXXI	$C_{75}H_{112}O_{36}$	1587.6855	Polygala
691	Onjisaponin F	$C_{75}H_{112}O_{36}$	1587.6855	Polygala
692	3-O-(β-D-Glucopyranosyl) presenegenin 28-(O-β-D-galactopyranosyl-(1→4)-O-[β-D-glucopyranosyl-(1→3)]-O-β-D-xylopyranosyl-(1→4)-O-α-L-rhamnopyranosyl-(1→2)-{4-O-[(E, Z)-4-methoxycinnamoyl]}-β-D-fucopyranosyl) ester	$C_{75}H_{112}O_{36}$	1587.6855	Polygala
693	Onjisaponin Gg	$C_{76}H_{112}O_{36}$	1599.6855	Polygala
694	Onjisaponin K	$C_{76}H_{112}O_{36}$	1599.6855	Polygala
695	Polygalasaponin XLIV	$C_{76}H_{114}O_{37}$	1617.6961	Polygala
696	Onjisaponin R	$C_{76}H_{114}O_{37}$	1617.6961	Polygala
697	3-O-(β-D-Glucopyranosyl) presenegenin 28-(O-β-D-galactopyranosyl-(1→4)-O-[β-D-glucopyranosyl-(1→3)]-O-β-D-xylopyranosyl-(1→4)-O-α-L-rhamnopyranosyl-(1→2)-{4-O-[(E, Z)-3,4-dimethoxycinnamoyl]}-β-D-fucopyranosyl)ester	$C_{76}H_{114}O_{37}$	1617.6961	Polygala
698	Polygalajaponicose I	$C_{75}H_{90}O_{40}$	1629.493	Polygala
699	E -Senegasaponin C	$C_{77}H_{114}O_{37}$	1629.6961	Polygala
700	Z -Senegasaponin C	$C_{77}H_{114}O_{37}$	1629.6961	Polygala
701	Onjisaponin O	$C_{77}H_{116}O_{37}$	1631.7117	Polygala
702	Polygalasaponin XL	$C_{74}H_{116}O_{40}$	1643.6965	Polygala
703	Polygalasaponin XLV	$C_{78}H_{116}O_{38}$	1659.7067	Polygala
704	Polygalasaponin XLVI	$C_{78}H_{116}O_{38}$	1659.7067	Polygala
705	Onjisaponin W	$C_{79}H_{116}O_{38}$	1671.7067	Polygala
706	Polygalasaponin XXXII	$C_{79}H_{118}O_{38}$	1673.7223	Polygala
707	Tenuifoside A	$C_{79}H_{118}O_{38}$	1673.7223	Polygala
708	Micranthoside C	$C_{75}H_{120}O_{41}$	1675.7227	Polygala
709	Polygalasaponin XLI	$C_{76}H_{118}O_{41}$	1685.7071	Polygala
710	Onjisaponin Ng	$C_{80}H_{118}O_{38}$	1685.7223	Polygala
711	OnjisaponinQg	$C_{76}H_{120}O_{41}$	1687.7227	Polygala
712	Myrtifolioside A1	$C_{79}H_{118}O_{39}$	1689.7172	Polygala
713	Myrtifolioside A2	$C_{79}H_{118}O_{39}$	1689.7172	Polygala
714	E-Senegin IV	$C_{80}H_{120}O_{39}$	1703.7329	Polygala
715	Z-Senegin IV	$C_{80}H_{120}O_{39}$	1703.7329	Polygala

716	Onjisaponin A	$C_{80}H_{120}O_{39}$	1703.7329	Polygala
717	OnjisaponinFg	$C_{81}H_{120}O_{40}$	1731.7278	Polygala
718	Onjisaponin S	$C_{81}H_{122}O_{40}$	1733.7434	Polygala
719	Onjisaponin Y	$C_{82}H_{122}O_{41}$	1761.7384	Polygala
720	Onjisaponin V	$C_{82}H_{122}O_{41}$	1761.7384	Polygala
721	Onjisaponin Vg	$C_{82}H_{122}O_{41}$	1761.7384	Polygala
722	Onjisaponin T	$C_{84}H_{128}O_{41}$	1791.7853	Polygala
723	Onjisaponin J	$C_{85}H_{126}O_{42}$	1817.7646	Polygala
724	Onjisaponin L	$C_{86}H_{128}O_{43}$	1847.7751	Polygala
725	Onjisaponin Sg	$C_{87}H_{130}O_{44}$	1877.7857	Polygala
726	Onjisaponin Ug	$C_{87}H_{130}O_{45}$	1893.7806	Polygala
727	Onjisaponin X	$C_{87}H_{130}O_{45}$	1893.7806	Polygala
728	OnjisaponinTg	$C_{90}H_{136}O_{45}$	1935.8276	Polygala
729	pinicolic acid A	$C_{30}H_{46}O_{3}$	453.68	Poriacocos
730	dehydropachymic acid	$C_{33}H_{50}O_{5}$	525.75	Poriacocos
731	Floralginsenoside A	$C_{42}H_{72}O_{16}$	831.4748	Ginseng
